# Generic Interpretable Reaction Condition Predictions with Open Reaction Condition Datasets and Unsupervised Learning of Reaction Center

**DOI:** 10.34133/research.0231

**Published:** 2023-10-16

**Authors:** Xiaorui Wang, Chang-Yu Hsieh, Xiaodan Yin, Jike Wang, Yuquan Li, Yafeng Deng, Dejun Jiang, Zhenxing Wu, Hongyan Du, Hongming Chen, Yun Li, Huanxiang Liu, Yuwei Wang, Pei Luo, Tingjun Hou, Xiaojun Yao

**Affiliations:** ^1^ Dr. Neher’s Biophysics Laboratory for Innovative Drug Discovery, State Key Laboratory of Quality Research in Chinese Medicine, Macau Institute for Applied Research in Medicine and Health, Macau University of Science and Technology, Macao, 999078, China.; ^2^ Innovation Institute for Artificial Intelligence in Medicine of Zhejiang University, College of Pharmaceutical Sciences, Zhejiang University, Hangzhou, 310058, China.; ^3^ Faculty of Applied Sciences, Macao Polytechnic University, Macao, 999078, China.; ^4^ College of Chemistry and Chemical Engineering, Lanzhou University, Lanzhou, 730000, China.; ^5^ CarbonSilicon AI Technology Co., Ltd, Hangzhou, Zhejiang310018, China.; ^6^ Center of Chemistry and Chemical Biology, Guangzhou Regenerative Medicine and Health Guangdong Laboratory, Guangzhou 510530, China.; ^7^ College of Pharmacy, Shaanxi University of Chinese Medicine, Xianyang, Shaanxi, 712044, China.

## Abstract

Effective synthesis planning powered by deep learning (DL) can significantly accelerate the discovery of new drugs and materials. However, most DL-assisted synthesis planning methods offer either none or very limited capability to recommend suitable reaction conditions (RCs) for their reaction predictions. Currently, the prediction of RCs with a DL framework is hindered by several factors, including: (a) lack of a standardized dataset for benchmarking, (b) lack of a general prediction model with powerful representation, and (c) lack of interpretability. To address these issues, we first created 2 standardized RC datasets covering a broad range of reaction classes and then proposed a powerful and interpretable Transformer-based RC predictor named Parrot. Through careful design of the model architecture, pretraining method, and training strategy, Parrot improved the overall top-3 prediction accuracy on catalysis, solvents, and other reagents by as much as 13.44%, compared to the best previous model on a newly curated dataset. Additionally, the mean absolute error of the predicted temperatures was reduced by about 4 °C. Furthermore, Parrot manifests strong generalization capacity with superior cross-chemical-space prediction accuracy. Attention analysis indicates that Parrot effectively captures crucial chemical information and exhibits a high level of interpretability in the prediction of RCs. The proposed model Parrot exemplifies how modern neural network architecture when appropriately pretrained can be versatile in making reliable, generalizable, and interpretable recommendation for RCs even when the underlying training dataset may still be limited in diversity.

## Introduction

As a cornerstone of modern science and technology, any advancement of our mastery in chemical synthesis may bear a profound impact on the development of downstream disciplines such as pharmacy, environmental science, energy industry, and materials science. For decades, scientists have been attempting to build reliable and convenient computer-aided synthesis planning (CASP) tools [1–22]. With the recent advancement of computing power, deep learning (DL) algorithms, and theoretical understanding of electronic structures and chemical reactions, some reliable CASP tools have been developed, and they could potentially enhance chemists’ productivity in synthesis planning. For instance, contemporary CASP tools can achieve similar performance of synthetic route planning to human experts for some complex natural products [1]. With the explosive growth of experimental chemical data in recent decades, it is anticipated that DL-assisted synthesis planning (DASP) tools will inevitably play a more crucial role in the digitized chemistry discovery. The combination of DASP and robotic synthesis platforms promises to eventually automate the pipeline of molecular discovery and optimization, starting from in silico synthetic route planning to autonomous experimental synthesis [23] in a closed loop. Despite these encouraging progresses, many existing DASP algorithms still face nontrivial challenges [24], obstructing their wider applications in the labs. Particularly, it is difficult to automatically assess the quality of machine-proposed synthesis plans. A key factor that undermines the quality of DASP is that existing algorithms cannot reliably recommend comprehensive reaction conditions (RCs) for a broad array of reactions as needed in organic syntheses.

**Fig. 1.**
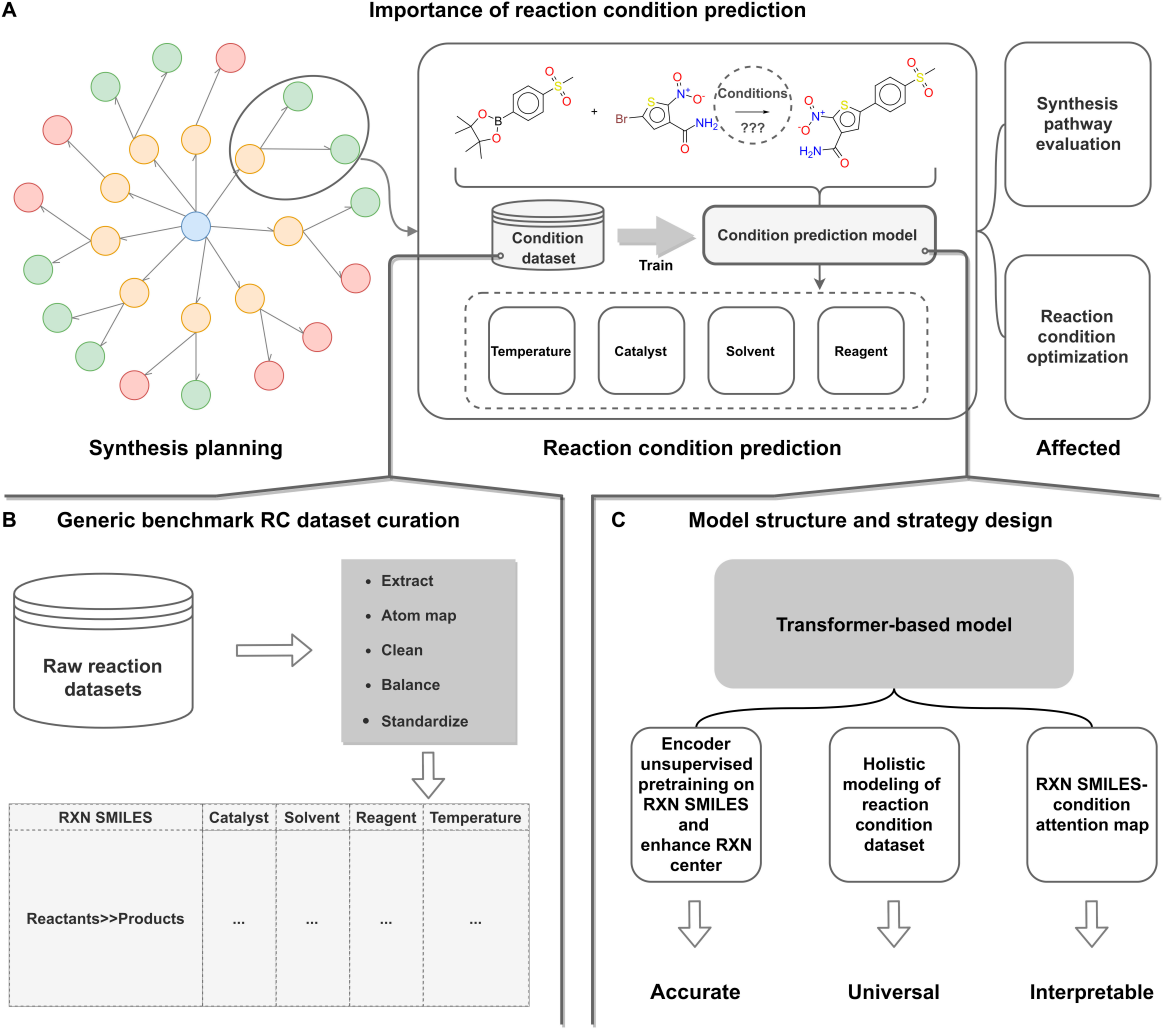
Overview. (A) Effects of RC prediction tasks on synthesis planning. (B) Schematic representation of the processing flow and structure of the RC dataset. (C) Parrot model structure and strategy design.

The choices of a reasonable chemical environment (catalysts, reagents, and solvents) and other operating conditions (temperature, pressure, etc.) for reactions are crucial as they collectively determine what product molecules to be expected along with reaction yields and rates. In the past, researchers would query the literature to learn how similar molecules were synthesized, and then they would apply similar reactions to obtain the target molecules. In this scenario, researchers tend to adopt the reported RCs for their synthesis plans instead of consulting a computational algorithm for recommendations. This practice restricts the choice of RCs and often turn out to be suboptimal choices. With the continuing curation of valuable reaction data and development of DL, there have been attempts to develop algorithms that can recommend RCs, thus potentially overcoming the aforementioned limitations. As shown in Fig. 1A, RC prediction also has an increasing impact on the evaluation of synthesis pathways and the optimization of chemical RCs [25,26]. However, the development of a general DL-based RC predictor remains a complex challenge that has rarely been addressed. Most existing RC predictors only focus on predicting certain aspects of RCs (such as only solvents or reagents) or modeling a specific type of reactions (such as Suzuki reactions or Negishi reactions). For example, Walker et al. [27] predicted the solvents for 4 types of reactions, and Shim et al. [28] predicted the RCs for Pd-catalyzed coupling reactions. One prominent factor that hinders the development of a general RC prediction model is the lack of high-quality and open-source standardized RC datasets. Previous works [25,27–30] obtained data from commercial or private databases, such as Reaxys. Because the training and testing data in most of these earlier works have not been disclosed to the public, it is difficult for later practitioners to build new models and then compare against previously published models under a fair setting. Clearly, there is an urgent need for establishing a more standardized and open-source benchmark for RC predictions in order to stimulate or facilitate further algorithmic development on this front. However, while existing open reaction database [31] is highly favorable to DASP, existing data sources such as United States Patent and Trademark Office (USPTO) reaction data for RC prediction tasks still require further data cleaning and standardization to produce a reasonably reliable chemical RC dataset.

Another intriguing issue for the development of RC prediction algorithms is to determine an effective combination of DL model and associated representation of chemical reactions in order to model the intrinsic correlations between different factors of RC. Some works [27–29,32] have represented chemical reactions by molecular/reaction fingerprints as the input to feed-forward neural network or traditional machine learning model, but these representation methods are not inherently compatible with the more advanced DL algorithms such as Transformer with the attention mechanisms, which may improve the interpretability and prediction accuracy. There is also a work [30] using molecular graph and graph neural network (GNN) as the representation method and DL model, respectively, for predicting the RCs on a small-scale reaction dataset. This method proposed by Maser et al. is suitable for RC predictions when facing a training set with small sample size and a fixed number of molecular graphs are involved in the reaction samples (for example, the reactions always involve 2 reactants and 1 product), but it is not suitable for modeling more complex large-scale general RC data. For modeling multiple classes of RCs, the simplest way is to model multiple classes of RCs separately [22], but this approach cannot render a model to truly learn the intrinsic correlation between RCs. Gao et al. [29] formulated RC recommendations as a sequential prediction task and used a method similar to the recurrent neural network such that the RCs predicted in the previous step are fed into the model as the input for the next step prediction, which better considered the relationship between the predicted RCs, but the deep neural network architecture adopted by Gao et al. still has room for improvement in terms of interpretability and accuracy. Finally, a standardized benchmark dataset for RC prediction is currently lacking, and only a limited number of attempts have been reported to try different machine learning models and representation methods for RC prediction. Thus, this field can be greatly benefited if more advanced DL models can be reported and serve as strong baselines to inspire further algorithmic development. As already mentioned, without specifying appropriate RCs, all the CASP predictions are impractical, especially for a futuristic self-driving laboratory.

In this study, we spent considerable amount of time to curate high-quality RC datasets for benchmark purposes and developed an end-to-end RC prediction model named Parrot based on Transformer and pretrain strategy. We regarded the problem of RC prediction as a causal sequence prediction problem and completed the classification for multiple conditions and the regression of temperatures. The contributions of this work can be succinctly summarized as follows:

1. We curated a large open-source dataset named USPTO-Condition based on the original USPTO reaction dataset [33] for benchmarking RC recommendation models. In addition, according to the specific data extraction strategy, another general RC dataset Reaxys-TotalSyn-Condition was also extracted from Reaxys for a comprehensive model evaluation. The general procedure regarding the curation of the benchmark RCs dataset is shown in Fig. 1B.
2. Leveraging the attention-based model architecture and training methodology specifically designed for enhanced reaction center, our method Parrot achieved an overall top-3 prediction accuracy of 2.64% and 13.44% higher than the RCs recommender (RCR) proposed by Gao et al. [26] for 2 large-scale general RC datasets mentioned above, respectively, and the temperature mean absolute error (MAE) was also reduced by about 4 °C. Figure 1C shows the model structure and strategy used by Parrot.
3. We also demonstrated that our method exhibits strong generalization ability, maintaining higher predictive accuracy and suffering less accuracy loss compared to RCR [26] when prediction across reaction space.
4. Finally, we utilized the attention mechanisms to illustrate the intrinsic correlation between the substructures in the reaction and the predicted catalysts and reagents. The model design of Parrot can capture reaction centers and characteristic functional groups well. The model interpretation provides more scientific insights.

## Results

### Overview of methods

We treat the condition prediction task in 2 parts. The first part is the prediction of the chemical context, which is treated as a causal multitask multiclassification problem involving catalysts, 2 solvents, and 2 reagents. This treatment is similar to the work of Gao et al. [29]. The second part is the prediction of temperature, which is treated as a regression problem. Bert [34] is employed as the encoder to embed the reaction information directly from the simplified molecular input line entry system (SMILES) [35–38] (reactants >> products) to an abstract latent space, and then this machine-readable representation of reactions will be used to predict the downstream tasks, such as chemical context condition and temperature.

### Dataset

**Fig. 2.**
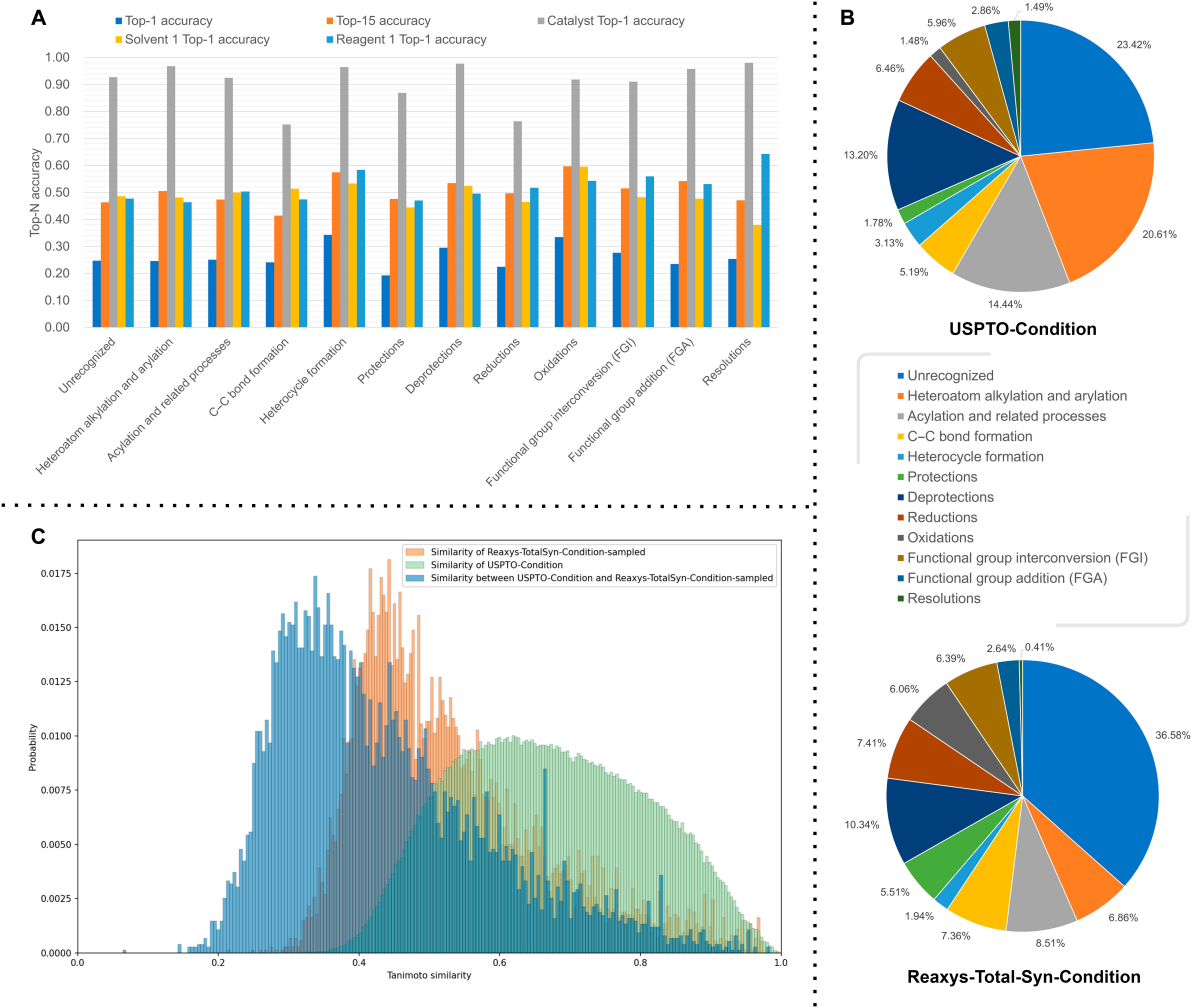
Visualization of the Parrot model prediction accuracy based on the reaction category. (A) Prediction performance based on the reaction category in the USPTO-Condition test set. (B) Reaction category composition of the USPTO-Condition dataset and Reaxys-TotalSyn-Condition dataset. (C) Distribution of the similarity between the reactions within USPTO-Condition and Reaxys-TotalSyn-Condition-Sampled and between USPTO-Condition and Reaxys-TotalSyn-Condition-Sampled.

We curated 2 large datasets, including USPTO-Condition (without temperature) and Reaxys-TotalSyn-Condition (with temperature), with the data volumes of 680,741 and 180,129, respectively. Both datasets were split according to the ratio of train:validation:test = 8:1:1 in this study. All molecules, such as the reactants and products in each entry of reaction, are recorded in canonical SMILES, and each data entry contains 1 reaction SMILES, temperature (in Reaxys-TotalSyn-Condition), and 5 chemical context labels, including catalyst, solvent1, solvent2, reagent1, and reagent2. For each class of chemical context condition (catalyst, solvent1, solvent2, reagent1, and reagent2), an additional “Null” category is added to represent that the reaction does not require this type of RC [29]. In the Reaxys-TotalSyn-Condition dataset, we retained the original details (RC name, concentration, etc.) of the chemical context conditions, implying that the model does not just predict the SMILES of chemical context condition molecules but needs to fully predict the conditions used in the original chemical reaction. The prediction task of the Reaxys-TotalSyn-Condition dataset is more difficult than USPTO-Condition due to the sparser RC labels. After completing the curation of these 2 datasets, we used a reaction classifier to classify the USPTO-Condition dataset and the Reaxys-TotalSyn-Condition dataset into 12 categories, respectively. The composition of the reaction categories for both datasets is shown in Fig. 2B, and the details about the reaction classifier can be found in Section S2.4. Finally, we also designed an external validation experiment to verify the prediction ability of the model across the chemical reaction space, where we extracted 8,413 reaction data (named Reaxys-TotalSyn-Condition-Sampled) from Reaxys that could be covered by the RC data labels and were significantly different from the USPTO-Condition dataset. The details of the processing methods for the USPTO-Condition dataset, Reaxys-TotalSyn-Condition dataset, and Reaxys-TotalSyn-Condition-Sampled test set are provided in Section S1, the processing scripts can be found at https://github.com/wangxr0526/Parrot, and the approach to obtain the curated RC datasets can be found in Data Availability. We have also summarized the key information of the USPTO-Condition and Reaxys-TotalSyn-Condition datasets in Tables S1 and S2, respectively.

### Model architecture

**Fig. 3.**
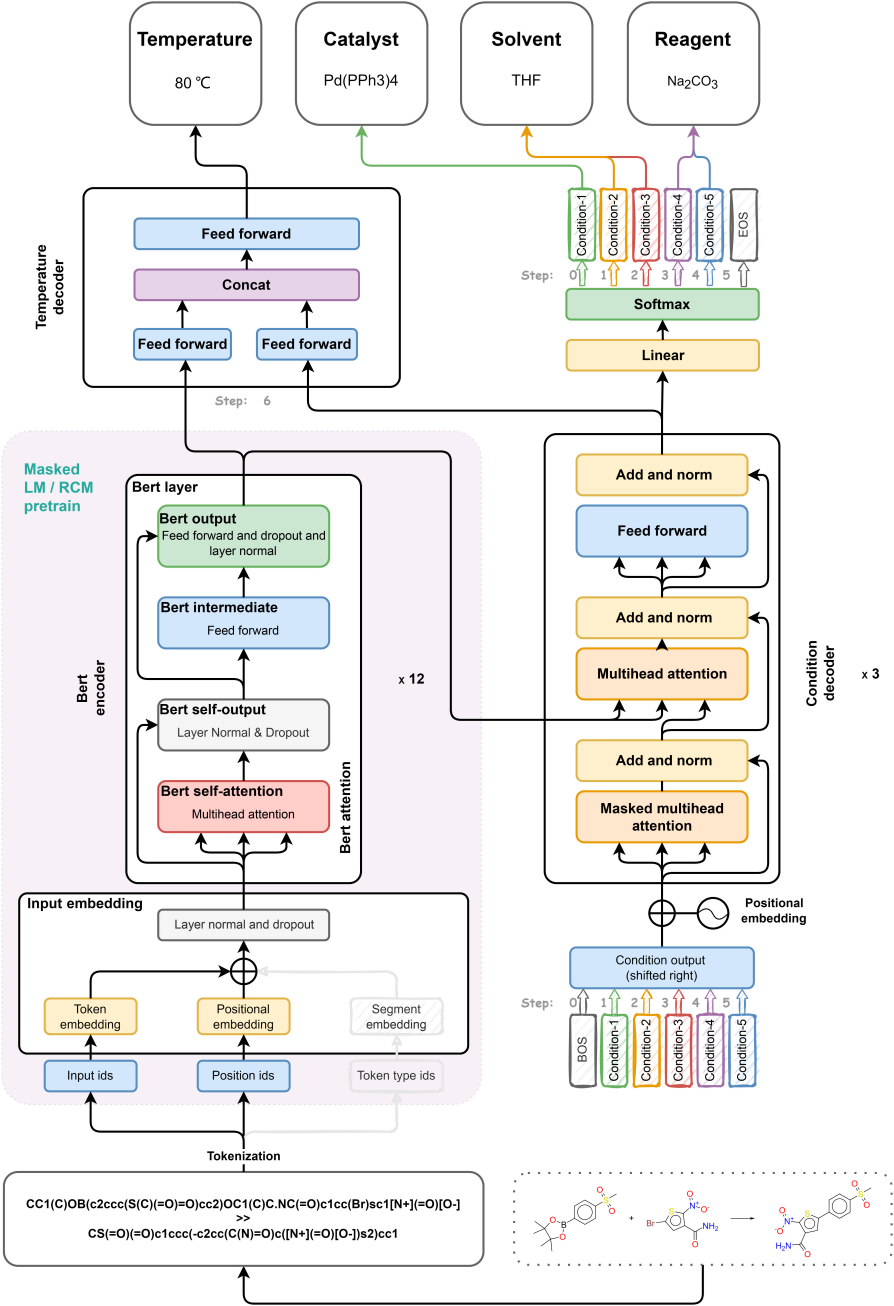
Parrot model architecture. The model decodes 5 contextual RCs in the first 5 steps of prediction. In the sixth step, it combines the tensor from the encoder and the tensor containing the information of the 5 contextual RCs from the condition decoder to predict the temperature.

The working principle of the DL models can be roughly conceptualized as a 2-stage process: autonomous feature learning and downstream task (classification, regression, etc.) predictions. Inspired by the natural language processing tasks and the works reported by Schwaller et al. [39–42], we proposed an interpretable pretrained reaction condition Transformer (Parrot). This model uses Bert-like encoder to extract the reaction features from SMILES and a Transformer decoder to generate the hidden-layer representation of reaction context conditions. Finally, the classifier is employed for sequential prediction of reaction context conditions, and the tensor containing 5 context condition information is combined with the reaction embedding tensor. This combined tensor is then passed through a regression layer, named temperature decoder, to estimate the temperature. Our model architecture is summarized in Fig. 3.

We treat the prediction of the chemical context (i.e., catalyst, solvent1, solvent2, reagent1, and reagent2) as a sequence to 5 condition multiclass classification tasks, and the conditions for postprediction also consider the conditions that have been predicted, with the target lengths fixed (*length* = 6). We use the information contained in the memory tensors from the encoder and the decoder output tensors toward the 5 RCs to predict the temperature. Each of these tensors is deformed by a feed-forward neural network, which is fed into a third feed-forward neural network to compute a scalar (temperature) after tensor concatenation.

The loss function we use consists of 2 parts, the classification part and the regression part. As the general sequence-to-sequence generation tasks, for the classification part we use cross-entropy as the loss function for the optimization of 5 conditions. For the regression part, we use mean squared error as the loss function. To balance the loss values between the regression and classification components, we introduced a coefficient α in the temperature regression loss. We tested various combinations of coefficients and ultimately determined that the optimal value for α is 0.001. The loss function equation is as follows:

$$\text{Loss}=\sum_{i\in I}\;\text{CrossEntropyLoss}\left(c_{i},{\hat{c}}_{i}\right)\;+\;\alpha *\text{MSELoss}\left(t,\hat{t}\right)$$ (1)

where *I* is the chemical context condition number, *c_i_* is the predicted label of the *i*-th condition, ${\hat{c}}_{i}$ is the ground truth label of the *i*-th condition, *t* is the predicted temperature, and $\hat{t}$ is the ground truth temperature. In our method, *I* = 6 (including 5 chemical context conditions and an end token). It is worth noting that when the temperature prediction function is not imposed on Parrot, the loss function only includes the classification loss function (the first part).

### Model pretrain strategy

The prediction of downstream tasks is deeply dependent on the embedding and representation of the source data. Inspired by the successful experiments on reaction classification and reaction yield prediction reported by Schwaller et al. [41,42], we also adopted a pretraining strategy when designing Parrot for RC predictions. Well-curated RC data with reaction classes and reaction yields is relatively rare, but there is a large inventory of raw chemical reaction data. In our pipeline, we also adopted a pretraining strategy to allow Bert (the encoder) to better embed reaction SMILES to hidden tensors through unsupervised learning.

We tried 2 pretraining strategies, i.e., masked language modeling (Masked LM) and masked reaction center modeling (Masked RCM) by incorporating the domain knowledge on chemical reactions. The reaction datasets we use in both pretraining strategies contain about 1.3 million reaction SMILES obtained by cleaning USPTO 1976-2016sep [33]. These data have been cleared of all RCs to include reactants and products only (reactants >> products) and keep the same format and content as the input of the RC prediction task. Considering the disparate distribution observed between the Reaxys-TotalSyn-Condition dataset and the USPTO-Condition dataset (as illustrated in Fig. 2B), alongside the relatively smaller size of the former, we developed a Masked RCM pretraining strategy aimed at acquiring domain knowledge related to reaction centers. With appropriate inductive bias, Parrot pretrained with Masked RCM delivers a superior performance when the training set is small. The schematic diagram of this strategy implementation is shown in Fig. S1. In the Masked RCM strategy, in order to strengthen the model's understanding of reaction centers, we increased the mask probability of the reaction center tokens to 0.5 instead of 0.15. The reaction center tokens were labeled by performing substructure matching, which involved matching reactions with their corresponding reaction templates using the rdkit [43] library. See Table S4 for the hyperparameters used in the Bert Masked LM and Masked RCM pretraining.

### Model performance

Unlike most of the previous works [27,28,30,32,44], our model is designed and trained for recommending RCs for generic scenarios. Since no specification of reaction types is required, Parrot can be directly embedded into exiting synthetic planning algorithms to determine the optimality of a given synthesis path. Our model is more versatile than models trained on a single type of reaction data. For a more comprehensive evaluation of our method, we cleaned 2 general RC datasets used for benchmarking, named USPTO-Condition and Reaxys-TotalSyn-Condition, extracted from USPTO and Reaxys, respectively.

#### USPTO-Condition results

For this dataset, we conducted 6 ablation experiments to investigate the influence of the presence or absence of pretraining, types of pretraining, and the size of the decoder (number of layers and number of heads) on model performance. We also conducted an enhanced training experiment for further improving the prediction accuracy of the Parrot model. The evaluated variants of Parrot in this dataset include Parrot-D, Parrot-LM, Parrot-RCM, Parrot-LM-E, Parrot-RCM-E, and Parrot-LM-6L8H. Parrot-D employed the strategy of initializing weights with a uniform distribution. For Parrot-LM and Parrot-RCM, the encoder weights were initialized using pretrained Masked LM and Masked RCM Bert models trained on the USPTO reaction dataset, respectively. To enhance prediction accuracy, we performed fine-tuning on ×5 SMILES augmented training set, which was created by 5-fold training data augmentation based on the weights of Parrot-LM and Parrot-RCM. These 2 enhanced models are referred to as Parrot-LM-E and Parrot-RCM-E, respectively. The performance of Parrot's 6 variants and the aforementioned baseline model RCR [29] on the entire test set is shown in Table 1. While solvent1 and reagent1 have a larger number of labels and a denser distribution, due to the sparse distribution of the labels for catalyst, solvent2, and reagent2, we adopted a strategy with fewer candidate selections for the sparse reaction categories of catalyst, solvent2, and reagent2. Conversely, for the dense condition categories of solvent1 and reagent1, we used a strategy with more candidate selections. In our evaluation on the USPTO-Condition dataset, our output condition strategy was to predict the top-1 catalyst, top-3 solvent1, top-1 solvent2, top-5 reagent1, and top-1 reagent2. Finally, the 15 results (combinations of all the RC predictions) were sorted according to the overall scores (softmax probability score product for each RC token), and the top-k accuracy was calculated. In this experiment, in order to compare the accuracy of each model more accurately, all top-k accuracies were calculated by imposing a strict matching. According to the results in Table 1, it can be seen that the catalyst (c) top-1 accuracies of all 7 models including the baseline model exceed 90%, but the accuracy of Parrot based on pretraining is higher than that of the RCR model. When using the Masked LM pretraining strategy (Parrot-LM), the top-1 accuracy reaches 92.35%, and with further enhanced training (Parrot-LM-E), the accuracy increases to 92.50%. The overall top-3 accuracy of Parrot-LM-E is 2.64% higher than that of the RCR model, and the top-15 accuracy improvement increases to 3.14%. Furthermore, except the top-1 accuracy for solvent2, Parrot-LM-E achieves the highest accuracy among all the model configurations we tried. In Parrot-D, Parrot-LM, Parrot-LM-E, Parrot-RCM, and Parrot-RCM-E, we employed 3 decoder layers with 4 attention heads per layer. We also tested the model configuration using 6 decoder layers and 8 attention heads per layer (named Parrot-LM-6L8H), and the impact on the results was very slight, indicating that this task is not sensitive to the decoder configuration. All of the subsequent experiments adopt the architecture of the decoder with 3 layers and 4 attention heads per layer. The model achieved the best accuracy when initialized with Masked LM parameters, as observed in Parrot-LM-E. Additionally, Parrot-RCM-E, initialized with Masked RCM, also achieved higher overall accuracy than RCR. Due to the larger size of the USPTO-Condition dataset, both pretraining methods had similar positive effects on downstream RC predictions, yielding excellent results. The difficulty of the RC predictions may vary among different reaction categories. We also examined the model performance by the chemical reaction category in this dataset. The accuracy of the Parrot-LM model for each reaction category is shown in Fig. 2A. We can see from Fig. 2A that Parrot-LM shows a relatively weak performance in predicting the C–C bond formation reaction in this dataset. Although AR-GCN proposed by Maser et al. [30] achieved excellent prediction accuracy for reactions such as Suzuki, Negishi, C–N couplings, and Pauson-Khand, there exists a substantial performance gap compared to the general RC prediction models RCR and Parrot in this dataset. Specifically tailored for small-scale datasets comprising a single reaction type, AR-GCN exhibited an overall top-1 accuracy that was approximately 10% lower than the generic RC prediction model on USPTO-Condition dataset. As a result, it may not be well suited for RC prediction tasks that are closer to the real-world applications. For the development of the GNN-based condition prediction models applied in the context of general synthetic planning, we also selected the work by Zhang et al. [22] (referred to as CIMG-Condition) as a comparison. Their approach involved modeling multiple RC strategies separately, and we established the CIMG-Condition prediction models for each condition category in the USPTO-Condition dataset. By employing the same inference strategy, we compared this method and found that the overall top-1 accuracy was approximately 7% lower compared to the models that consider the interdependencies between conditions (RCR and Parrot). Due to significant differences among the modeling strategies used by their approach, our method and the RCR model, we have included the performance of the CIMG-Condition models in the Supplementary Information for readers' reference. For detailed accuracy information on USPTO-Condition dataset of AR-GCN and CIMG-Condition models, please refer to Table S10.

**Table 1.** Results of the Parrot model on the USPTO-Condition test set and comparison with the baseline model^a^.

Models	Chemical context condition accuracy↑					
	Conditions	Top-1	Top-3	Top-5	Top-10	Top-15
RCR [29]	c	0.9219	0.9219	0.9219	0.9219	0.9219
	s1	0.5015	0.6640	0.7055	0.7340	0.7346
	s2	0.8130	0.8369	0.8461	0.8525	0.8527
	r1	0.4972	0.6597	0.7402	0.8184	0.8516
	r2	0.7622	0.8408	0.8664	0.8876	0.8986
	Overall ^b^	0.2596	0.3771	0.4206	0.4612	0.4717
Parrot-D	c	0.9196	0.9196	0.9196	0.9196	0.9196
	s1	0.4712	0.6557	0.7027	0.7273	0.7278
	s2	0.8093	0.8361	0.8440	0.8494	0.8495
	r1	0.4759	0.6511	0.7387	0.8249	0.8620
	r2	0.7558	0.8375	0.8660	0.8911	0.9029
	Overall	0.2397	0.3672	0.4159	0.4596	0.4723
Parrot-LM	c	0.9235	0.9235	0.9235	0.9235	0.9235
	s1	0.4927	0.6772	0.7228	0.7462	0.7468
	s2	0.8067	0.8421	0.8511	0.8566	0.8568
	r1	0.4961	0.6727	0.7569	0.8393	0.8740
	r2	0.7648	0.8413	0.8717	0.8948	0.9052
	Overall	0.2576	0.3893	0.4386	0.4813	0.4934
Parrot-LM-E	c	0.9250	0.9250	0.9250	0.9250	0.9250
	s1	0.5018	0.6858	0.7311	0.7536	0.7543
	s2	0.8096	0.8426	0.8521	0.8582	0.8585
	r1	0.5039	0.6820	0.7629	0.8436	0.8776
	r2	0.7648	0.8486	0.8774	0.8998	0.9110
	Overall	0.2691	0.4035	0.4510	0.4914	0.5031
Parrot-LM-6L8H ^d^	c	0.9228	0.9228	0.9228	0.9228	0.9228
	s1	0.4889	0.6755	0.7220	0.7456	0.7462
	s2	0.8062	0.8406	0.8500	0.8562	0.8565
	r1	0.4936	0.6743	0.7583	0.8405	0.8763
	r2	0.7608	0.8476	0.8762	0.9000	0.9106
	Overall	0.2574	0.3902	0.4373	0.4811	0.4929
Parrot-RCM	c	0.9224	0.9224	0.9224	0.9224	0.9224
	s1	0.4837	0.6671	0.7118	0.7369	0.7377
	s2	0.8071	0.8405	0.8500	0.8571	0.8573
	r1	0.4871	0.6663	0.7513	0.8343	0.8696
	r2	0.7612	0.8428	0.8701	0.8949	0.9067
	Overall ^b^	0.2529	0.3841	0.4318	0.4728	0.4848
Parrot-RCM-E ^c^	c	0.9240	0.9240	0.9240	0.9240	0.9240
	s1	0.4929	0.6756	0.7204	0.7434	0.7441
	s2	0.8073	0.8426	0.8525	0.8585	0.8587
	r1	0.4956	0.6767	0.7577	0.8405	0.8751
	r2	0.7626	0.8463	0.8752	0.8980	0.9095
	Overall	0.2621	0.3947	0.4417	0.4829	0.4951

^a^ c, s1, s2, r1, and r2 refer to catalyst, solvent 1, solvent 2, reagent 1, and reagent 2, respectively.

^b^ Overall: c, s1, s2, r1, and r2.

^c^ E: This model is fine-tuned for 2 epochs with a small learning rate using a 5× data augmentation training set based on LM/RCM.

^d^ 6L8H: The decoder of the default Parrot model is 3 layers and 4 heads per layer, and 6 layers and 8 heads per layer are used in this experiment.

#### Reaxys-TotalSyn-Condition results

Unlike USPTO-Condition that only has the chemical context condition data, the Reaxys-TotalSyn-Condition dataset gives the operating temperature for each reaction. On this dataset, we conducted the 2 experiments and compared the effect of the type of the pretraining strategies on the Parrot model accuracy. Regarding the evaluation of the chemical context conditions prediction, we used the same method as USPTO-Condition to calculate the top-k accuracy. In addition, we also used MAE as an evaluation method for temperatures. For the prediction of temperatures, we used the decoder hidden tensor corresponding to the chemical context condition top-1 as part of the temperature decoder input. In this dataset, many reaction records do not use catalyst, solvent2, and reagent2, and these items rarely show up. Especially for catalyst, we found that 96% of all records has no catalyst in the test set. In order to evaluate the performance of the model more reasonably, we divided the test set into 2 parts, namely, the part containing the catalyst (denoted as Alpha group) and the part not containing the catalyst (denoted as Beta group). The model evaluation method described in Experiment details. The performances of these models in this dataset are shown in Table 2. In the Alpha part of the test results, the Parrot-RCM model achieved the highest accuracy, with an overall (s1r1) top-1 accuracy of 7.76% higher than the RCR model, and top-15 accuracy expanded to 13.70%. In the Beta part of the test, which contains less data, the Parrot-RCM model also achieved a higher overall (c1s1r1) top-1 accuracy of 9.7% than the RCR model, and top-15 accuracy expanded to 18.16%. The temperature MAE of both parts (Alpha and Beta) was reduced by about 4 °C compared to that of the RCR model. Comparing these 2 pretraining strategies, the RCM pretraining strategy performs better than LM in Reaxys-TotalSyn-Condition. The accuracy of AR-GCN on this general RC dataset still remains significantly lower compared to RCR and Parrot. Due to the lack of consideration for the relationships between RCs, the prediction accuracy of the general RC prediction model CIMG-Condition is lower than those of RCR and Parrot. However, it is noticeably superior to AR-GCN, which is specifically designed for modeling small datasets. The specific accuracy values on Reaxys-TotalSyn-Condition of AR-GCN and CIMG-Condition models are included in Table S11.

**Table 2.** Results of the Parrot model on the Reaxys-TotalSyn-Condition test set and comparison with the baseline model^a^.

Models	Chemical context condition accuracy↑						Temperature MAE↓
	Conditions	Top-1	Top-3	Top-5	Top-10	Top-15	
Alpha: Test results for the portion of the test set without catalyst ^b^							
RCR	s1	0.5664	0.6873	0.7226	0.7683	0.7700	25.87
	r1	0.4141	0.5506	0.6143	0.6650	0.6896	
	overall(s1r1)	0.3102	0.4597	0.5135	0.5598	0.5761	
Parrot-RCM	s1	0.5931	0.7739	0.8090	0.8291	0.8298	21.67
	r1	0.5114	0.6834	0.7508	0.8160	0.8423	
	overall(s1r1)	0.3878	0.5812	0.6424	0.6957	0.7131	
Parrot-LM	s1	0.5855	0.7656	0.8039	0.8241	0.8245	22.17
	r1	0.5011	0.6747	0.7454	0.8147	0.8424	
	overall(s1r1)	0.3775	0.5691	0.6341	0.6952	0.7131	
Beta: Test results for the portion of the test set containing the catalyst ^c^							
RCR	c1	0.1866	0.3134	0.3433	0.4900	0.4900	24.77
	s1	0.3806	0.4975	0.5647	0.6244	0.6468	
	r1	0.2662	0.4403	0.5100	0.6791	0.7388	
	Overall (c1s1r1)	0.0746	0.1517	0.1940	0.2612	0.2985	
Parrot-RCM	c1	0.2861	0.4552	0.5050	0.6194	0.6194	20.39
	s1	0.4229	0.6095	0.7040	0.7910	0.8159	
	r1	0.3980	0.6269	0.7040	0.8333	0.8706	
	Overall (c1s1r1)	0.1716	0.2861	0.3557	0.4453	0.4801	
Parrot-LM	c1	0.2189	0.4080	0.4851	0.6493	0.6493	21.97
	s1	0.3806	0.5871	0.6915	0.7587	0.7687	
	r1	0.3259	0.5672	0.6741	0.8308	0.8682	
	Overall (c1s1r1)	0.1045	0.2537	0.3259	0.4527	0.4826	

^a^ c, s1, s2, r1, and r2 refer to catalyst, solvent 1, solvent 2, reagent 1, and reagent 2, respectively.

^b^ Portfolio of predicted results: s1 top3 and r1 top5; the amount of data in this part is 17,611.

^c^ Portfolio of predicted results: c1 top2, s1 top3, and r1 top5; the amount of data in this part is 402.

### Generalizable prediction capabilities across reaction space

To evaluate the Parrot model's ability across reaction space prediction, we created an external test set for the model trained on USPTO-Condition, called Reaxys-TotalSyn-Condition-Sampled. This external test set was derived from the Reaxys-TotalSyn-Condition dataset, and we selected a portion of the dataset with RC labels that can be covered by USPTO-Condition as the external test set. The production process of this part of the dataset is introduced in Section S1.3. In order to quantify the distribution difference between the Reaxys-TotalSyn-Condition-Sampled dataset and USPTO-Condition dataset in chemical space, we calculated the average similarity of each chemical reaction to its 5 most similar reactions within and between these 2 datasets, respectively. The similarity distribution histogram is visualized in Fig. 2C. The reaction data are represented using the reaction difference fingerprint (calculated from extended connectivity fingerprints [45]) and the similarity calculation method is tanimoto similarity. The blue distribution histogram shows a significant chemical space difference between the external test set Reaxys-TotalSyn-Condition-Sampled and the training set USPTO-Condition. Differences in the distribution between the training and test sets can significantly increase the challenges in model prediction. It can be used to assess the ability of different models to predict across reaction spaces. For the detailed information on the calculation method of the similarity analysis used in this section, please refer to Section S6.

In this experiment, we used the same test approach as Reaxys-TotalSyn-Condition to calculate the accuracy based on whether the test set contains catalysts, with the Alpha part containing 7,428 test data without catalysts and the Beta part containing 202 test data with catalysts. Since the solvents and reagents in the RCs are quite substitutable, we also adopted a more relaxed metrics for evaluation. The idea is that if the predicted results for solvent and reagent match the substitutable part (i.e., belonging to the same category) of the ground truth, then the predictions are considered to be correct for both types of chemical RCs. The classification method of solvents refers to the solvent similarity index [46], and the classification method of reagents is based on the key substructure fingerprint. The detailed classification method is introduced in Sections S3.1 and S3.2. The test results of the Parrot-LM-E and the baseline model RCR in the cross-chemical space predictions experiment are shown in Table 3.

**Table 3.** Test results of Parrot and RCR when predicting across the chemical reaction space.

Models	Dataset information		Chemical context condition accuracy↑ ^d^					
	Train	Test	Conditions ^a^	Top-1	Top-3	Top-5	Top-10	Top-15
Alpha: Test results for the portion of the test set without catalyst ^b^								
RCR	USPTO ^e^	Sampled ^f^	s1	0.4962	0.6334	0.673	0.7131	0.7138
			r1	0.4654	0.6080	0.6781	0.7535	0.7882
			Overall (s1r1)	0.2977	0.4335	0.4894	0.5487	0.5696
RCR	USPTO	USPTO	s1	0.5919	0.7333	0.7709	0.7981	0.7987
			r1	0.5321	0.6885	0.762	0.8359	0.8675
			Overall (s1r1)	0.3814	0.5376	0.6026	0.6721	0.6959
Parrot-LM-E ^g^	USPTO	Sampled	s1	0.5127	0.6695	0.7122	0.7402	0.7407
			r1	0.5162	0.6517	0.7189	0.7994	0.8335
			Overall (s1r1)	0.3438	0.4814	0.5408	0.6062	0.6282
Parrot-LM-E	USPTO	USPTO	s1	0.5900	0.7565	0.7984	0.8202	0.8209
			r1	0.5407	0.7115	0.7871	0.8604	0.8926
			Overall (s1r1)	0.3873	0.5654	0.6367	0.7077	0.7325
Beta: Test results for the portion of the test set containing the catalyst ^c^								
RCR	USPTO	Sampled	c1	0.1683	0.2426	0.3317	0.4554	0.4554
			s1	0.2871	0.4802	0.5545	0.6139	0.6535
			r1	0.3168	0.4505	0.5446	0.6683	0.7277
			Overall (c1s1r1)	0.0248	0.0545	0.104	0.2228	0.2822
RCR	USPTO	USPTO	c1	0.6263	0.7074	0.7527	0.8171	0.8171
			s1	0.5549	0.6847	0.7447	0.7946	0.8172
			r1	0.5307	0.6655	0.7441	0.8091	0.8451
			Overall (c1s1r1)	0.2769	0.3986	0.467	0.543	0.579
Parrot-LM-E	USPTO	Sampled	c1	0.1832	0.2574	0.3317	0.4505	0.4505
			s1	0.2921	0.5495	0.6089	0.6733	0.7030
			r1	0.2871	0.5297	0.6040	0.7376	0.7673
			Overall (c1s1r1)	0.0792	0.1634	0.2228	0.3218	0.3614
Parrot-LM-E	USPTO	USPTO	c1	0.6144	0.7094	0.7508	0.8209	0.8209
			s1	0.5448	0.7091	0.7726	0.8244	0.8447
			r1	0.5360	0.6882	0.7608	0.8301	0.8650
			Overall (c1s1r1)	0.2839	0.4177	0.4845	0.5692	0.6038

^a^ c1, s1, and r1 refer to catalyst, solvent 1, and reagent 1, respectively;

^b^ Portfolio of predicted results: s1 top3 and r1 top5; the amount of data in this part is 7,428.

^c^ Portfolio of predicted results: c1 top2, s1 top3, and r1 top3; the amount of data in this part is 202.

^d^ Top-k accuracy is calculated using the close math of solvents and reagents.

^e^ USPTO: USPTO-Condition dataset.

^f^ Sampled: Reaxys-TotalSyn-Condition-Sampled.

^g^ E: This model is fine-tuned for 2 epochs with a small learning rate using 5× data augmentation training set based on LM.

In the Alpha part of the test results, when the test data was switched from the USPTO test set, which had the same training data, to the Reaxys-TotalSyn-Condition-Sampled test set with a significantly different data distribution from USPTO, both the Parrot and RCR models experienced varying degrees of accuracy reduction. RCR suffered a more notable decrease, with the top-1 accuracy of s1r1 dropping from 38.14% to 29.77%, resulting in an 8.37% decrease in accuracy. On the other hand, Parrot-LM-E demonstrated more robust performance, experiencing only a 4.35% decrease. Similar observations were made in the Beta part of the test results. Although there are significant differences in reaction similarity between the Reaxys-TotalSyn-Condition-Sampled test set and the USPTO-Condition training set, the Parrot model achieved better prediction accuracy than RCR. We further show the differences in the decrease in accuracy of the various reaction types after transitioning the test set from USPTO-Condition to Reaxys-TotalSyn-Sampled in Section S8 of the Supplementary Information. These results indicate that the Parrot model exhibits significantly higher cross-chemical space prediction capability compared to the RCR model. The Parrot's stronger ability to predict across reaction spaces is contributed from its cross-attention mechanism's enhanced learning capability regarding the relationship between reaction features and RCs. Additionally, the pretraining strategy further enhances the learning of reaction features. This allows the Parrot model to perform well even in situations where there are significant differences in the chemical space of the dataset, enabling it to capture crucial information effectively.

### Interpretability results

In this section, we conducted analyses from 2 different perspectives to explore the information embedded in the attention mechanism of the Parrot model when predicting RCs. In the first analysis, we investigated the model’s understanding of crucial reaction centers. In a different analysis, we delved deeper into the correlation between the predicted RCs and the functional groups present in the inputted reactions. Through these 2 analyses, we gain a more comprehensive understanding of the performance and information extraction capabilities of the Parrot model in predicting chemical RCs. Finally, we also visualized some reaction cases as demonstrations.

#### Analysis results of the Parrot's understanding of reaction centers

**Fig. 4.**
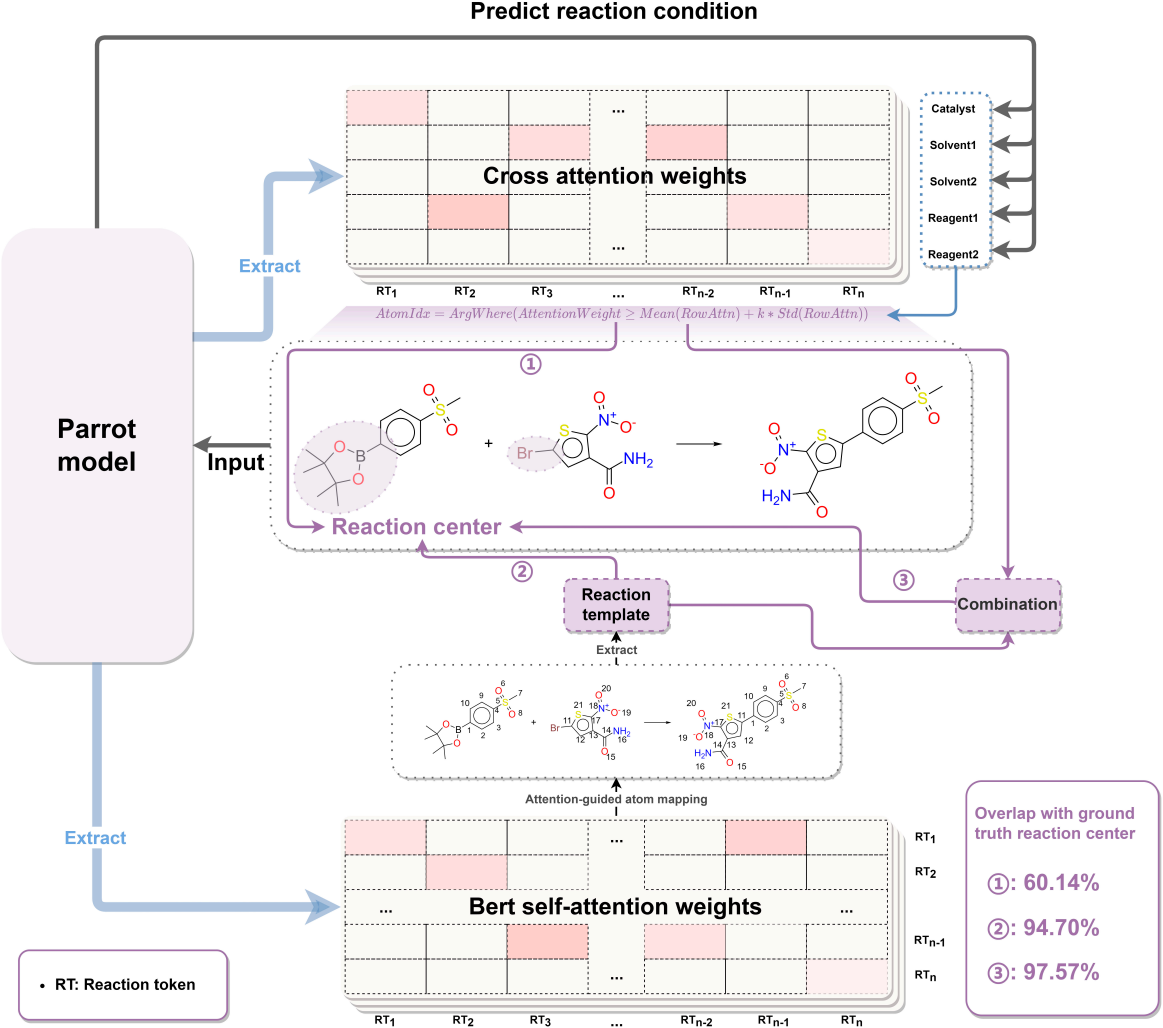
Reaction center analysis schematic diagram. In the Parrot model, while predicting chemical RCs, the cross-attention weights and the encoder's self-attention weights are extracted to obtain reaction center information using 3 methods: ① Using only the cross-attention mechanism, potential active atoms that may be reaction centers are determined by setting a threshold using the validation set. ② Using only the encoder's self-attention mechanism, an atom mapping algorithm guided by self-attention weights is used to label the atom mapping between reactants and products, followed by extracting reaction templates to identify potential active atoms that may be reaction centers. ③ Both the cross-attention mechanism and the encoder's self-attention mechanism are considered simultaneously to determine potential active atoms that may be reaction centers.

In this part of the analysis, we employed 3 strategies to investigate the attention mechanism of the Parrot model. These strategies involved examining the cross-attention mechanism, the self-attention mechanism of the encoder, and a comprehensive analysis of both attention mechanisms. We evaluated the model’s understanding of reaction centers by comparing the overlap score (OS) between the selected active atoms represented by cross-attention weights and self-attention weights with the ground truth reaction centers. The schematic diagram of the method pipeline is shown in Fig. 4. During the analysis process, we introduced certain parameters that were adjusted on the USPTO-Condition validation set, and the final results were obtained on the USPTO-Condition test set. Table 4 presents the OS, false positive rate (FPR), and accuracy of the reaction centers for the 3 attention information extraction strategies (cross-attention, Bert self-attention, and combination). The accuracy of the reaction centers was assessed using 2 criteria. The first criterion, “half”, considered active atoms overlapping with at least half of the reaction center atoms as hits, while the second criterion, “at least 2”, required active atoms to overlap with at least 2 reaction center atoms to be classified as hits. Further details of the analysis can be found in Interpretability analysis.

**Table 4.** The OS, FPR, and accuracy of the reaction centers for the 3 attention information extraction strategies in terms of the active atoms and reaction centers.

Dataset	Attention type	OS	FPR	Reaction center accuracy	
				Half	At least 2
Val	Cross attention	60.36%	32.79%	71.14%	94.29%
Test		60.14%	32.96%	70.57%	94.61%
Val	Bert self-attention	94.77%	9.44%	95.13%	95.31%
Test		94.70%	9.49%	95.10%	95.28%
Val	Combination	97.60%	14.68%	98.35%	99.70%
Test		97.57%	14.67%	98.38%	99.75%

According to the results shown in Table 4, the following observations can be made: The OS between the active atoms indicated by the cross-attention mechanism and the reaction centers is relatively low at 60.14%. The FPR is 32.96%. The accuracy of the reaction centers is 70.57% (half) and 94.61% (at least 2). This suggests that the cross-attention weights not only focus on the information of the reaction centers but also seem to capture other important information for RCs prediction task. In subsequent interpretability analysis, we demonstrate that this portion of important information captured by the cross-attention weights is closely associated with a nonreactive characteristic functional group. In contrast, the active atoms identified by the Bert self-attention mechanism exhibit a higher OS with the reaction centers, reaching 94.70%. The FPR is low at only 9.49%. The accuracy of the reaction centers is 95.10% (half) and 95.28% (at least 2). This indicates that the encoder of the Parrot model demonstrates a remarkable understanding of the reaction center information. In the third analysis approach, by combining the information from the cross-attention mechanism and the encoder's self-attention mechanism, the OS between the active atoms and the reaction centers is further increased to 97.57%. The accuracy of the reaction centers significantly improves, reaching 98.38% (half) and 99.75% (at least 2). This suggests that the Parrot model exhibits complementary advantages in understanding the reaction centers under different attention mechanisms. Furthermore, we also observe that these 4 metrics demonstrate consistent performance across the test and validation sets, which to some extent validates the reliability of our analysis approach. However, solely attending to the information of the reaction centers is insufficient for better predicting RCs. In order to further explore the information beyond the reaction centers that the cross-attention mechanism focuses on, we have designed an alternative perspective for analysis.

#### Analysis results of attention-based association between functional groups and RCs

**Fig. 5.**
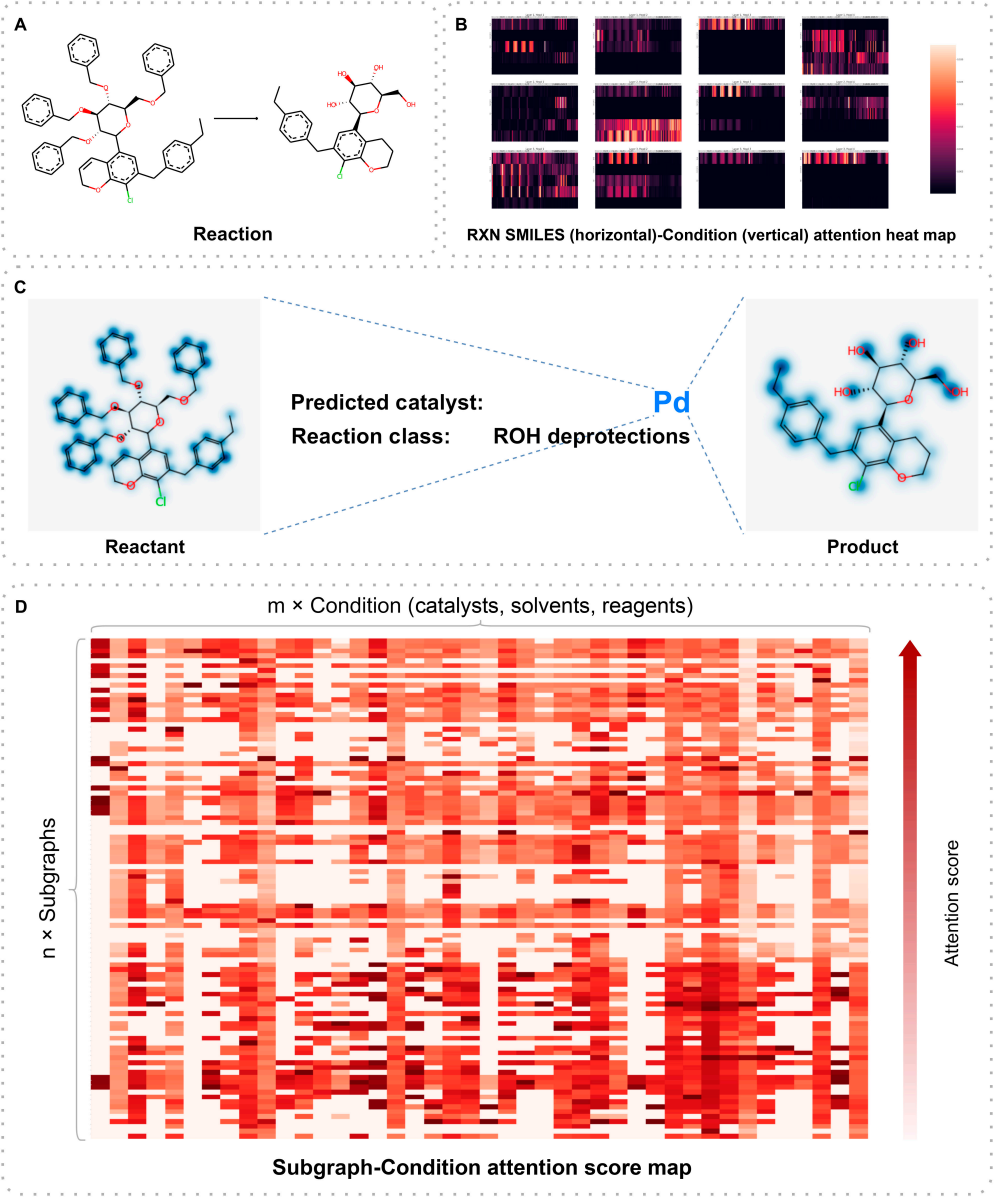
Example visualization of the reaction-condition attention weights. (A) Reaction example from the USPTO-Condition test set. (B) Brief visualization of the attention weights of the encoder’s memory by the decoder’s 3-layer 4 attention heads, with each subgraph having reaction SMILES horizontally and 5 contextual conditions vertically. (C) After averaging the attention weights of each attention head in each layer and visualized on the molecule, the attention weights with the palladium catalyst are shown here. (D) Brief visualization of the subgraph-Condition ASM (shown here is the Subgraph-Catalyst ASM). See Data Availability for source data table.

In this analysis, we utilized cross-attention weights to investigate the relationship between reactions and predicted RCs, focusing on the level of functional groups. Firstly, we selected a palladium-catalyzed alcohol deprotection reaction as an example, visualized in Fig. 5A to C. Figure 5B presents the heat map of attention weights, displaying the correspondence between key subsequences in the reaction SMILES and the predicted RCs (high-resolution images of this example can be found in Figs. S2 to S13). Figure 5C illustrates the attention weights of palladium catalyst relative to the atoms in the reactants and products, clearly indicating a higher attention on the atoms involved in the reaction center. However, there are still some cases where the attention weight distribution is challenging to interpret. Hence, in order to comprehensively investigate the grasp of group information by the cross-attention mechanism, we employed a macroscopic approach to analyze the attention distribution at the group level across the entire USPTO-Condition test dataset. We employed the BRICS [47] algorithm for reaction functional group segmentation and calculated the average attention weights across multiple heads and layers. Detailed analysis methods can be found in Interpretability analysis. Through this analytical approach, we obtained correlation scores based on attention weights for different catalysts, solvents, and reagents with respect to various chemical functional groups. We refer to these correlation score matrices as attention score maps (ASMs). Figure 5D displays the ASM between chemical group substructures and the catalyst, with similar ASMs obtained for solvents and reagents. However, due to space limitations, we are unable to present the ASMs for all RCs in main text. Please refer to Data Availability for the tables containing ASMs.

**Fig. 6.**
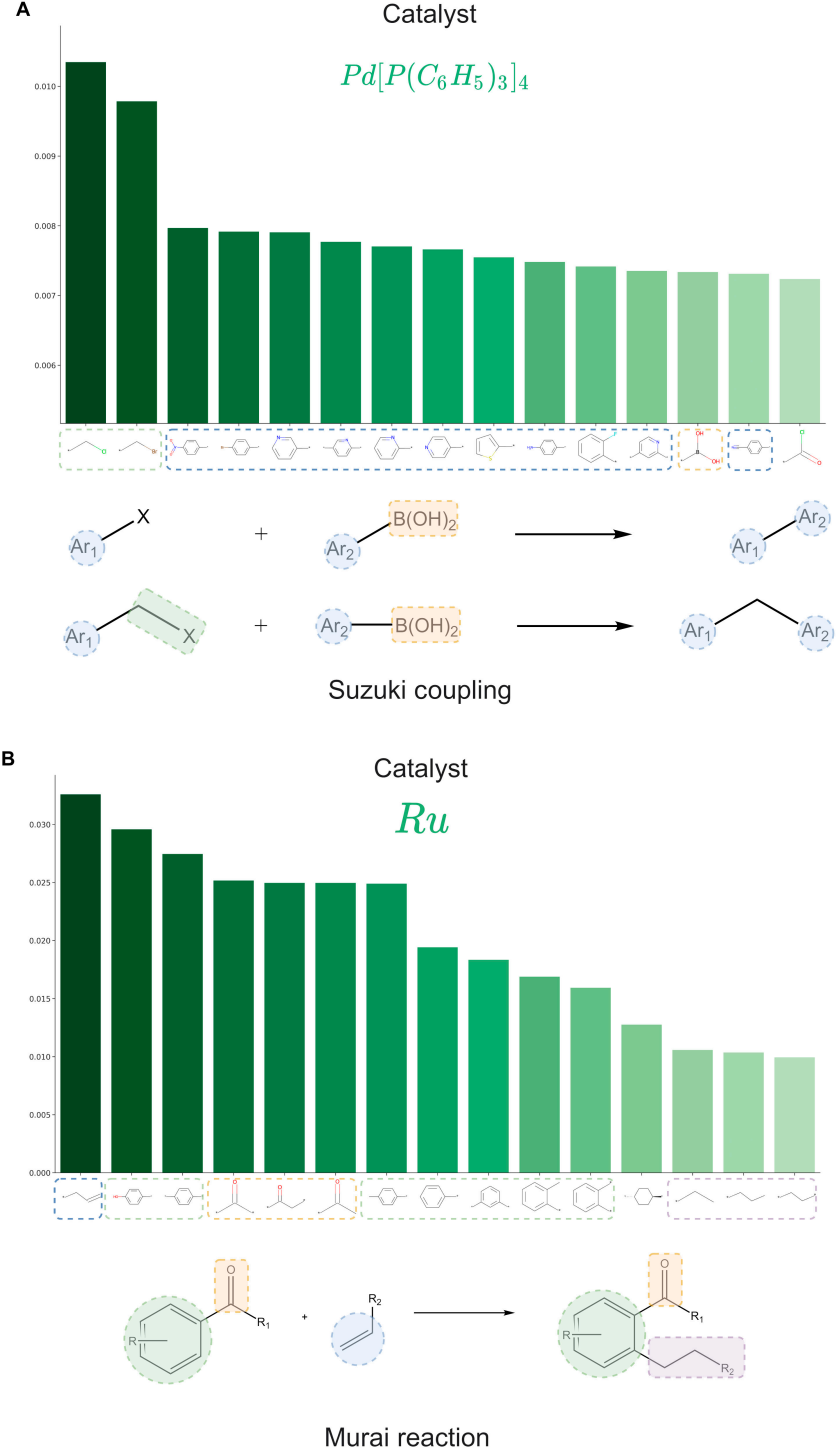
Catalysts and their highly correlated molecular subgraphs in the ASM.

**Fig. 7.**
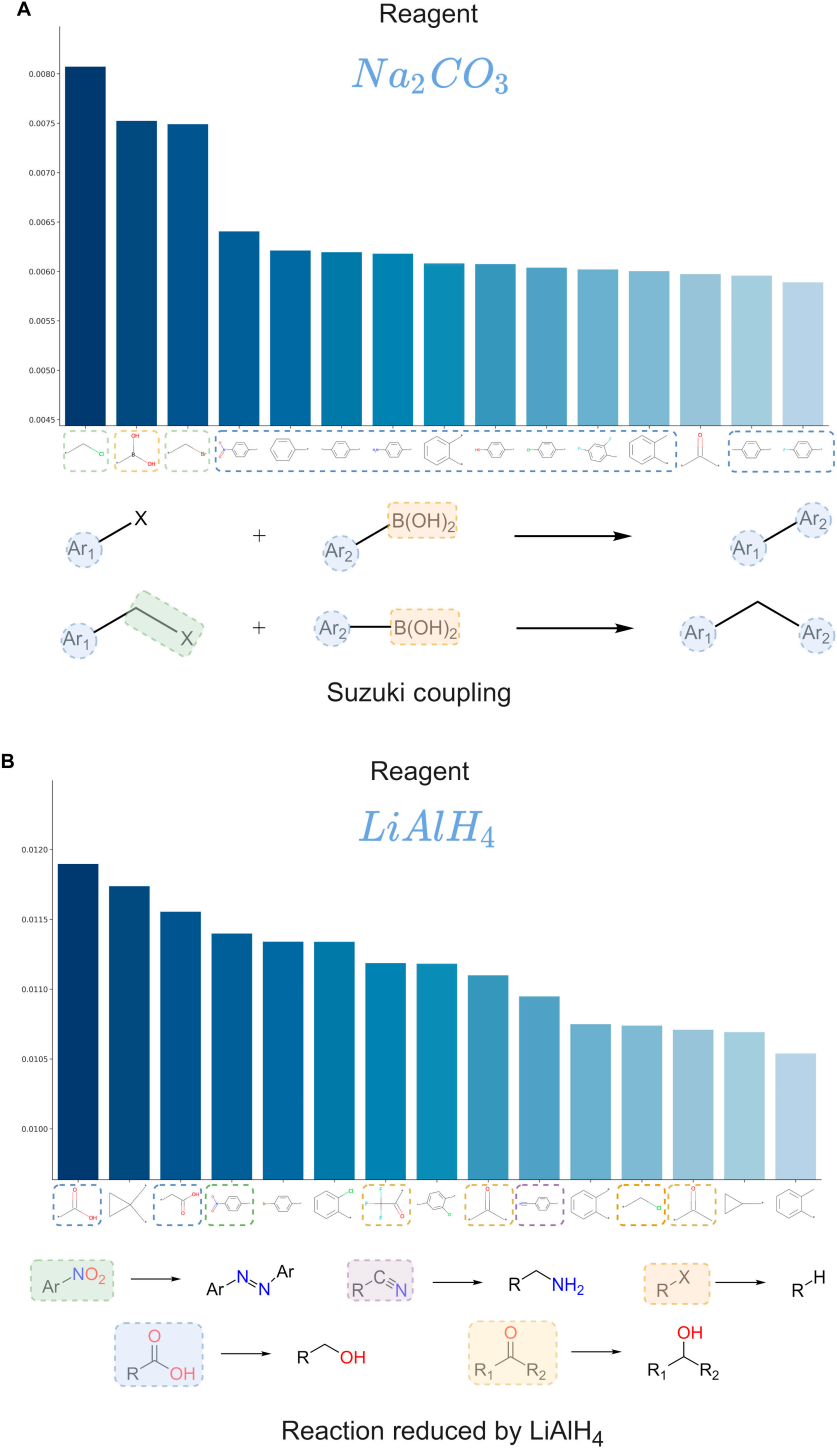
Reagents and their highly correlated molecular subgraphs in the ASM.

According to the ASM sorted by column (condition), multiple molecular substructures most relevant to individual RCs can be identified. We further visualized the molecular substructures for each RC (catalyst, solvent, and reagent) sorted by attention scores. The results revealed that the high attention score substructures for catalysts and reagents corresponded to characteristic molecular structures involved in the reactions. Two typical examples are visualized in Fig. 6. Figure 6A displays tetrakis(methyldiphenylphosphine) palladium and its top 15 relevant molecular substructures. Among these 15 substructures, 11 are aromatic groups (highlighted by blue boxes) and the first 2 are halogenated groups (highlighted by green boxes). These substructures are characteristic groups for the Suzuki reaction catalyzed by this catalyst. Another example is shown in Fig. 6B, representing substructures related to a ruthenium metal catalyst. The most relevant groups in this case are terminal alkene structures (highlighted by blue boxes), and among the top 6 related substructures, there are also 3 important carbonyl structures (highlighted by orange boxes). Additionally, among the top 15 substructures, 7 contain aromatic structures with benzene rings (highlighted by green boxes). These substructures are characteristic groups for the Murai reaction catalyzed by the ruthenium catalyst. The reaction reagents also exhibit high correlation scores with characteristic reaction substructures. Two typical examples of the reagents are visualized in Fig. 7, where Fig. 7A displays the top 15 molecular groups most relevant to sodium carbonate (base). We can observe that the top 3 ranked groups are chlorinated, boronic acid, and brominated groups, and the remaining highly correlated molecular groups are aromatic groups, which perfectly aligns with the Suzuki reaction. Figure 7B displays the top 15 molecular groups most relevant to lithium aluminum hydride (reducing agent), which is commonly used as a reducing agent in organic reactions. The highly correlated groups shown in the figure are those that can be reduced by lithium aluminum hydride and include carboxyl, nitro, cyano, carbonyl, and halogenated groups. Since solvents do not directly participate in the reaction, their ASM representation does not exhibit the same level of prominence as catalysts and reagents directly involved in the reaction. These examples demonstrate how the Parrot model automatically learns the relationship between reactions and predicted RCs (particularly catalysts and reagents) through attention mechanisms, thus exhibiting strong interpretability

#### Interpretability case study

**Fig. 8.**
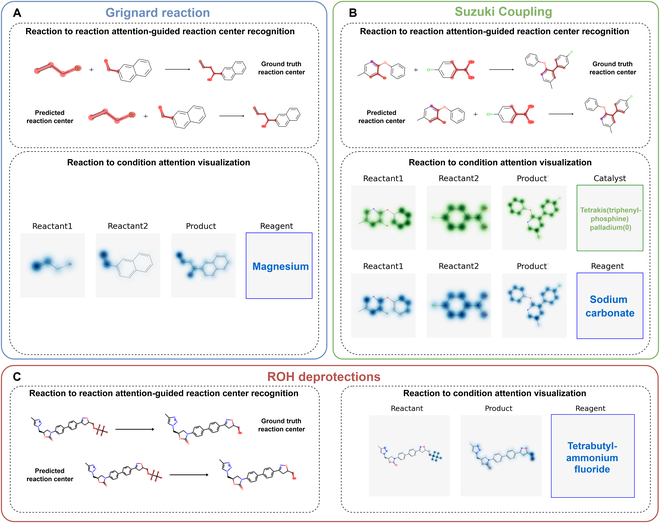
Visualizations of reaction information identified by Parrot’s two attention mechanisms, showcased in case studies of (a) Grignard reactions, (b) Suzuki coupling reactions, and (c) hydroxyl deprotection reactions. Each case visualizes two parts of information: 1) the model’s identification results of reaction centers, and 2) the reaction functional groups attended by the model when predicting reaction conditions. In the visualization of the model’s identification results of the reaction center, the reaction center is highlighted in red, with the top row indicating the ground truth reaction center and the second row representing the predicted reaction center by the Parrot model. In the visualization of attention weights related to reaction conditions, the catalyst and its associated groups are highlighted in green, with darker shades indicating higher weights. Reagent1 and its associated groups are highlighted in blue.

In this subsection, we visualized some cases of Parrot's predicted RCs. Figure 8 displays the reaction centers and typical functional groups that the model's attention mechanism focused on when predicting 3 types of reactions: Grignard reaction, Suzuki coupling reaction, and alcohol deprotection reaction. Each reaction type is highlighted with a different color frame. Each visualization case consists of 2 parts: (a) identification of reaction centers guided by the self-attention weights of the Parrot model's encoder and (b) functional group structures that the cross-attention weights between the encoder and the decoder of the Parrot model focus on when predicting catalyst or reagent. In these case studies, we employed the parameter configurations that demonstrated the best performance for the reaction center recognition task. In all 3 cases, the reaction centers identified by the self-attention weights of the Parrot model's encoder accurately matched the real reaction centers. In the Grignard reaction case presented in Fig. 8A, the Parrot model assigned high attention weights to the aldehyde group of reactant2 and the hydroxyl group of the product when predicting the magnesium metal as a reagent. It also placed relatively high attention weights on the bromine substitution structure of reactant1. These functional groups are typical for Grignard reactions. In the Suzuki coupling reaction case depicted in Fig. 8B, the model's attention was not only focused on the boronic acid group and the bromo substituent when predicting the metal palladium catalyst tetrakis(triphenylphosphine)palladium(0), but it also exhibited significant attention weights toward the aromatic ring. This indicates that the model's attention is not limited to the reaction center alone. Additionally, the model displayed less attention weights toward ether bonds or nitrogen atoms on aromatic rings, which are less relevant to the Suzuki coupling reaction. A similar phenomenon was observed when the model predicted sodium carbonate as reagent. In the alcohol deprotection reaction case illustrated in Fig. 8C, the model highly attended to the protecting group on the reactant and the hydroxyl group on the product. These cases clearly demonstrate the interpretability of the Parrot's 2 attention mechanisms when predicting RCs.

### Limitation and outlook

Although 2 RC datasets have been curated in this work, it should be noted that the existing datasets still face some limitations:

1. The dataset USPTO-Condition is obtained from the US patent database, which exhibits certain limitations in terms of data quality. Specifically, the accuracy of the recorded reaction temperatures is subject to notable errors. Therefore, we did not include temperature information in USPTO-Condition.
2. Similar to the work of Gao et al. [29], this study also adopts the strategy of constructing a model to predict 5 categories of RCs, and (following their workflow) we omit rare data with more than 5 RCs in these 2 datasets.
3. Although this work has demonstrated that Parrot has stronger cross-reaction space prediction capabilities compared to other similar models for predicting RCs, similar to other works that treat RC prediction as a classification task, Parrot's predictions heavily rely on the quality of collected RC data. For novel RCs that are not present in the dataset, the model struggles to make accurate predictions. The approach of decoding RCs step by step at the character level can overcome the limitations of RC labels. However, it also brings the issues related to syntax effectiveness and the assignment of roles to RCs.
4. We did not take into account of the high and low chemical yields when curating the data and training the model. In other words, the model considered all combinations of RCs present in the dataset to be of equal value. However, the chemical reaction yields are strongly affected by the RCs.
5. Although this study made efforts to clean the RC data obtained from USPTO and Reaxys, the data size is still limited compared to large-scale commercial datasets. This limitation is also evident in the trained models' performance. Access to higher-quality and larger-scale RCs data can improve RC prediction model performance further. Presently, large language models such as GPT-4 [48,49] have excelled in literature summarization. Employing similar techniques, it is feasible to automatically extract chemical RC data from extensive chemical synthesis literature to enhance data quality.

As part of the future research, we plan to integrate the chemical reaction yield into the development of chemical RC prediction models. Our proposed model for chemical reaction prediction can be seamlessly integrated into existing synthesis planning algorithms, thereby aiding in the optimization of synthesis routes.

## Discussion

In this study, we address the RC prediction task, which is essential for synthesis planning and RC optimization. In response to the lack of readily available open-source datasets for the RC prediction task, we curated 2 general RC datasets named USPTO-Condition and Reaxys-TotalSyn-Condition, which contain approximately 680,000 and 180,000 RC data, respectively. Here, we also proposed a novel RC prediction model called Parrot, which achieved the best performance on both datasets by incorporating a pretraining strategy using reaction domain knowledge and a well-designed training pipeline. Compared with the baseline model, the Parrot model improved the overall top-3 accuracy of reaction context condition prediction by 2.64% and 13.44%, respectively, and the MAE of the temperature prediction was reduced by about 4 °C. Our proposed model can not only simultaneously predict multiple types of RCs for multiple chemical reactions but also provide good interpretability by using an attention mechanism to gain insight into the intrinsic relationship between the molecular substructures in reactions and RCs. Additionally, Parrot can be seamlessly integrated into existing synthesis planning algorithms, offering synthesis chemists improved capabilities in designing reaction routes and optimizing RCs. Moreover, our open-source code includes a user-friendly web-based graphical user interface (GUI), providing convenient access for researchers to utilize Parrot's functionalities. Looking ahead, with the availability of a larger volume of high-quality RCs data, we anticipate that Parrot and Parrot-inspired algorithms will emerge as indispensable components of DASP tools, effectively guiding the development of self-driving laboratories.

## Methods

### Software and implementation

All the codes were implemented in the python, the rdkit [43] cheminformatics toolkit was used for data processing, and the model was constructed based on the pytorch [50] library. The web-based GUI was implemented using flask [51] library. The Parrot model is trained on Dell Precision 7920 Tower (Intel Xeon Bronze 3204, NVIDIA Quadro RTX8000 GPU, 512 GB RAM), and it can be inferred on a consumer computer Dell OptiPlex 7090 (Intel Core i7-11700, 8 GB RAM) without discrete GPU.

### Experiment details

We choose RCR [29] that also predicts multiple chemical RCs at the same time as the baseline model to compare with Parrot. In the later discussion, we report the RCR’s performance on the 2 datasets we prepared under the same dataset split. RCR is a model that uses the reaction fingerprints as the input of the feed-forward neural network to predict RCs.

For the prediction of the reaction context conditions, we adopted the top-k accuracy evaluation scheme to compare models, and for the temperature prediction, we adopted the MAE. During the test, we took the predictions of the USPTO-Condition test set for the first-ranked catalyst, the top-3 solvent1, the first-ranked solvent2, the top-5 reagent1, and the first-ranked reagent2. Finally, all predictions are sorted by the product of the logistic scores to compute the top-k accuracy. Since roughly 96% of the reaction records in the Reaxys-TotalSyn-Condition dataset do not use catalyst (i.e., the entry has null value in that column), we divided the test set into 2 separate categories depending on whether a catalyst is present in the reaction record. This gives us a more precise characterization of the models’ performance on RC predictions. The first test set contains the data without catalyst, and we predicted the top-3 solvents1 and the top-5 reagents1; the second test set contains the data with catalyst, and we predicted the top-2 catalysts, the top-3 solvents1, and the top-3 reagents1. The number of top candidates predicted for each RC category is determined by the sparsity level of the corresponding condition labels. Sparse labels result in fewer candidate selections (smaller top-k values), such as catalyst, solvent2, and reagent2. Conversely, dense labels lead to a larger number of candidate selections (larger top-k values), as seen in solvent1 and reagent1. For an illustrative diagram of the top-k accuracy calculation process for RC prediction across all models, please refer to Fig. S15.

We employed various model weight initialization schemes to train the Parrot model. For the USPTO-Condition dataset, we trained Parrot-D employed the strategy of initializing weights with a uniform distribution. Parrot-LM with encoder parameters initialized from a pretrained Masked LM model on the USPTO reaction dataset, and Parrot-RCM with encoder parameters initialized from a pretrained Masked RCM model on the USPTO reaction dataset. To further improve the prediction accuracy of Parrot, we also utilized the enhanced training method of Parrot-LM-E and Parrot-RCM-E, which involved a ×5 augmentation of the training set. This enhanced training method included data augmentation through SMILES permutation and multiple reactants (and products) shuffling and initialized the model parameters from the trained Parrot-LM or Parrot-RCM, followed by additional training for 2 epochs with a lower learning rate (1 × 10^−6^). For the Reaxys-TotalSyn-Condition dataset, we performed the Masked RCM initialization model training Parrot-RCM and Masked LM initialization model training Parrot-LM. We also trained and tested the GNN-based RC prediction model, AR-GCN, proposed by Maser et al., which is based on GNNs. This comparison aimed to assess the performance differences between a general RC prediction model and a model specifically designed for small-scale, specific types of RC datasets in large-scale prediction tasks. Furthermore, another graph-network-based generic RC prediction model, CIMG-Condition, is also included in the comparison. To ensure fair comparison, all models were trained using the same dataset splitting method, and the hyperparameters of each model were optimized during training to achieve optimal performance. See Section S2.2 for training details and hyperparameter selection of RCR, AR-GCN, CIMG-Condition, and Parrot.

### Interpretability analysis

Many previous works have demonstrated that attention mechanism can capture key chemical reaction information [52–54], but the interpretability in RC prediction is rarely studied. In this section, we utilized several attention-based methods to analyze and demonstrate how the Parrot RC prediction model captures the relationship between the details of chemical reactions and the predicted RCs. We analyzed the self-attention weights of the encoder (Bert) component as well as the cross-attention weights between the encoder and the RC decoder. These 2 sets of attention weights respectively manifest the model's understanding of the relationships among various atoms in the reaction and the model's understanding of the associations between the functional groups in the reaction and each RC.

#### Analysis of the Parrot's understanding of reaction centers

We first analyzed the model’s understanding of the reaction center, which is the most distinctive part of a chemical reaction. As shown in Fig. 4, we extracted the cross-attention weights (representing the relationship between reaction atoms and conditions) and the self-attention weights of Bert (representing the relationship between reactant and product atoms) when the Parrot model predicts the conditions. Next, we utilized the extracted cross-attention matrix and self-attention matrix separately and employed the following approach to analyze them on the USPTO-Condition validation set. We attempted 3 methods to establish correspondences between the information embedded in the attention weights and the reaction center:

1. Using only the reaction-condition cross-attention weights:

First, we normalize the cross-attention weight score vector corresponding to each RC and select the active atoms using the mean and standard deviation. The selected atoms follow the following formula:

$$\text{ActiveAtomIdx}=\text{ArgWhere}\left({\text{Attn}}_{C}\geq \text{Mean}\left({\text{Attn}}_{C}\right)+k*\mathrm{Std}\left({\text{Attn}}_{C}\right)\right)$$ (2)

In this formula, *ActiveAtomIdx* represents the indices of atoms selected as active atoms. *Attn* is an attention weight vector that represents the attention values of the chemical reaction SMILES sequence for a specific RC (catalyst, solvent1, solvent2, reagent1, and reagent2). *Mean*(•) denotes the average of attention weights for a specific RC, while *Std*(•) represents the standard deviation. *k* is a constant used to control the number of active atoms. It is multiplied by the standard deviation and added to the mean to determine the threshold for attention weights, which is used to select active atoms. If an atom's attention weight is greater than or equal to this threshold, it is considered an active atom. It is important to note that the cross-attention weights contain matrices for multiple layers and heads, so the aforementioned calculations need to be performed for each layer and head matrix. The hyperparameters include *Layer*, *Head*, *k*, and the ConditionType (*C*), which are optimized on the validation set.

The evaluation criterium consists of 2 components: the OS and the FPR. The optimal parameters are determined by maximizing the difference between OS and FPR. The calculation method is as follows:

$$\mathrm{TP}=\mathrm{len}\left(\text{ActiveAtomIdx}\cap \text{ReactionCenterAtomIdx}\right)$$ (3)

$$\mathrm{TP}+\mathrm{FN}=\mathrm{len}\left(\text{ReactionCenterAtomIdx}\right)$$ (4)

$$\mathrm{FP}=\mathrm{len}\left(\text{ActiveAtomIdx}\;\Delta \;\text{ReactionCenterAtomIdx}\right)$$ (5)

$$\mathrm{FP}+\mathrm{TN}=\mathrm{len}\left(\text{ActiveAtomIdx}\right)$$ (6)

$$\mathrm{OS}=\frac{\mathrm{TP}}{\mathrm{TP}+\mathrm{FN}}$$ (7)

$$\mathrm{FPR}=\frac{\mathrm{FP}}{\mathrm{FP}+\mathrm{TN}}$$ (8)

$${\text{ArgMax}}_{\text{Layer},\text{Head},k,C}\left(\mathrm{OS}-\mathrm{FPR}\right)$$ (9)

Here, TP represents the number of active atoms that match the ground truth reaction center atoms, TP+FN represents the total number of ground truth reaction center atoms, FP represents the number of active atoms falsely predicted as reaction center atoms, and FP+TN represents the total number of active atoms. The ground truth reaction center atoms are obtained by matching the reaction template subgraph corresponding to the reaction.

2. Using only Bert's reaction–reaction self-attention weights:
  First, the self-attention weight matrices extracted from Bert's encoder are inputted into the atom mapping algorithm implemented in rxnmapper [52]. To determine the optimal layer and head configuration, a methodology similar to that employed by Schwaller et al. [52] is adopted, using a dataset of 996 instances derived from the USPTO-50K. Subsequently, the atom mapping procedure is applied to the validation set of the USPTO-Condition, wherein the self-attention weights of Parrot's encoder serve as a guiding mechanism. The reaction templates are extracted using rdchiral [55]. Following that, rdkit is utilized to execute subgraph matching on the reactions, enabling the identification of indices associated with active atoms. Finally, a comparison is made between the indices of active atoms and the ground truth reaction center, upon which the OS and FPR are computed.

$$\text{ActiveAtomIdx}={\text{AtomIdx}}_{\text{TemplateMached}}$$ (10)

3. Simultaneously using cross-attention weights and Bert's self-attention weights:
  In this approach, we combine the cross-attention weights with the self-attention weights of the encoder to enhance the relevance between the attention mechanism and the reaction center. However, we adopt a stricter treatment for the cross-attention weights, and the calculation process is as follows:

$$\begin{array}{c} {\text{ActiveAtomIdx}}_{\text{cross}}=\\ \left(\text{ArgWhere}\left({\text{Attn}}_{\mathrm{CT}}\geq \text{Mean}\left({\text{Attn}}_{C}\right)+k*\mathrm{Std}\left({\text{Attn}}_{C}\right)\right)\right)\cup {\text{ArgTop}}_{n}\left({\text{Attn}}_{C}\right) \end{array}$$ (11)

$${\text{ActiveAtomIdx}}_{\text{self}}={\text{AtomIdx}}_{\text{TemplateMached}}$$ (12)

$$\text{ActiveAtomIdx}={\text{ActiveAtomIdx}}_{\text{cross}}\cup {\text{AtomIdx}}_{\text{TemplateMached}}$$ (13)

The first part of the calculation process involves obtaining the indices of active atoms using the cross-attention mechanism, denoted as *ActiveAtomIdx_cross_*. It consists of 2 components: the first part is the same as Method 1, and the second part involves identifying the top-ranked atoms based on attention weights, where the value of “*n*” is optimized on the validation set.

The second part involves obtaining the indices of active atoms using the self-attention mechanism, denoted as *ActiveAtomIdx_cross_*. These indices are obtained through template matching, similar to the second method, where the template is marked by an atom mapping algorithm guided by the self-attention weights of Parrot's encoder and extracted using rdchiral [55].

Finally, the indices of active atoms obtained from the cross-attention mechanism and the self-attention mechanism are merged to obtain the final indices of active atoms, represented as *ActiveAtomIdx*. The calculation of the OS and FPR for the obtained active atoms follows the same approach as Method 1. Seven parameters are optimized on the validation set: the constant *k*, the ConditionType (*C*), the *Head* and *Layer* for the cross-attention part, the *Head* and *Layer* for the self-attention part, and the constant *n*.

The parameter optimization space and the optimal parameters for the 3 aforementioned parts can be found in the Section S2.6. The attention weights used for calculating active atoms are normalized separately for reactants and products.

#### Analysis of attention-based association between functional groups and RCs

Furthermore, we conducted further exploration of the cross-attention weights between chemical reactions and RCs to analyze the additional information contained within them, apart from the reaction center information. In the analysis described in this part, we first fragmented all molecules (reactants and products) from USPTO reaction records using BRICS [47], counted the number of occurrences for the fragments, and selected the most representative and important 103 substructures for analysis. Then, in the test set of USPTO-Condition, we counted the attention weights calculated by the model according to the substructure and then calculated the attention map according to the number of the hits of the substructure. The calculation goes as follows:

$$A_{i,j}=\text{Mean}\left(\left\{S_{1}^{\mathrm{GC}},S_{2}^{\mathrm{GC}},\ldots ,S_{E}^{\mathrm{GC}}\right\}\right)$$ (14)

$$S_{e}^{\mathrm{GC}}=\frac{\sum_{w\in G}a_{e}}{n}$$ (15)

where *A* is the attention map matrix; *i* and *j* are the indices of substructure *G* (from reactants and products) and condition *C*, respectively; *E* is the number of times that substructure *G* is matched in the dataset; and *a_e_* is the average attention weight (by heads) connecting atom *w* to chemical context condition *C* (*e*th hit). *n* is the number of the atoms in substructure *G*. The results of this analysis can be found in Results. The detailed calculation process of the attention weight *a_e_* can be found in Section S2.3.

## Data Availability

USPTO-Condition dataset is freely available at https://github.com/wangxr0526/Parrot (link1). Reaxys-TotalSyn-Condition requires a subscription license as it comes from Reaxys, but we provide a complete data extraction process and processing scripts, which are also available at https://github.com/wangxr0526/Parrot/tree/master/preprocess_script/reaxys_script (link2). Figure 5D source data table is available at https://github.com/wangxr0526/Parrot/blob/master/paper_data/USPTO_condition_frag_analysis_results/c1_score_map.xlsx (link3). ASM source tables of catalyst, reagent, solvent are available at https://github.com/wangxr0526/Parrot/blob/master/paper_data/USPTO_condition_frag_analysis_results (link4); catalyst ASM: c1_score_map.xlsx, reagent1 ASM: r1_score_map.xlsx. All high-resolution figures in the manuscript can be found at https://github.com/wangxr0526/Parrot/tree/master/paper_data/paper_figure (link5).

## Supplementary Materials

Supplementary 1 Supplementary Information 1: Sections S1 to S8, Figs. S1 to S16, and Tables S1 to S11. Supplementary Information 2.
