# Supplementary material for: Generic Interpretable Reaction Condition Predictions with Open Reaction Condition Datasets and Unsupervised Learning of Reaction Center
Source: Research (Wash D C). 2023 Oct 16;6:0231. doi: 10.34133/research.0231 (PMC10578430; doi:10.34133/research.0231)
Supplement: Supplementary 1 — Supplementary Information 1: Sections S1 to S8, Figs. S1 to S16, and Tables S1 to S11. Supplementary Information 2. [file research.0231.f1.zip › supplementary_information-revision3.docx]

Supplementary Information for “**Generic Interpretable Reaction Condition Predictions with Open Reaction Condition Datasets and Unsupervised Learning of Reaction Center”**

Xiaorui Wang^†,€,#^, Chang-Yu Hsieh^*,‡,#^, Xiaodan Yin^†,€^, Jike Wang ^‡,€^, Yuquan Li^§^, Yafeng Deng^€^, Dejun Jiang^‡,€^, Zhenxing Wu^‡,€^, Hongyan Du^‡^, Hongming Chen^δ^, Yun Li^§^, Huanxiang Liu ^ø^, Yuwei Wang ^ะ^, Pei Luo^†^,

Tingjun Hou^*,‡^, Xiaojun Yao^*ø^

^†^Dr. Neher’s Biophysics Laboratory for Innovative Drug Discovery, State Key Laboratory of Quality Research in Chinese Medicine, Macau Institute for Applied Research in Medicine and Health, Macau University of Science and Technology, Macao, 999078, China.

^‡^Innovation Institute for Artificial Intelligence in Medicine of Zhejiang University, College of Pharmaceutical Sciences, Zhejiang University, Hangzhou, 310058, China.

^ø^Faculty of Applied Sciences, Macao Polytechnic University, Macao, 999078, China.

^§^College of Chemistry and Chemical Engineering, Lanzhou University, Lanzhou, 730000, China.

^€^CarbonSilicon AI Technology Co., Ltd, Hangzhou, Zhejiang 310018, China.

^δ^Center of Chemistry and Chemical Biology, Guangzhou Regenerative Medicine and Health Guangdong Laboratory, Guangzhou 510530, China.

^ะ^College of Pharmacy, Shaanxi University of Chinese Medicine, Xianyang, Shaanxi, 712044, China.

^#^These authors contributed equally to this work.

^*^**Corresponding authors:**

**Xiaojun Yao**

**E-mail:** xjyao@mpu.edu.mo.

**Tingjun Hou**

**E-mail:** [tingjunhou@zju.edu.cn](mailto:tingjunhou@zju.edu.cn).

**Chang-Yu Hsieh**

**E-mail:** kimhsieh@zju.edu.cn.

**1. Dataset Curation**

**1.1 USPTO-Condition**

We curated the USPTO-Condition from the USPTO raw dataset through the following steps:

1. Select the most 1976~2016 grants and 2001~2016 application .xml files from the original USPTO[1] data, and use the text processing method to extract patent source, reaction SMILES, catalysts and solvents originally marked in the dataset. After this step of extraction, we obtained 3,130,812 raw data.
2. Using rxnmapper to reassign the atom map to the original reaction SMILES, all compounds that do not participate in product atomic contributions and are not in the catalysts and solvents originally recorded are classified as "reagents". Subsequently, the duplicate data was further removed, and 1,117,867 data were obtained.
3. Ionic reagents are identified and data containing erroneous reagents is deleted. Items (catalyst, solvent, reagent) with less than 100 occurrences were deleted, and items with a number of catalysts greater than 1 and a number of solvent reagents greater than 2 were deleted. The remaining data after this step is 680741.
4. The data set is randomly divided into the training set, the validation set and the test set, with a ratio of 8:1:1 (544591:68075:68075). All data containing the same reaction are classified into the training set, and the validation set and test set only retain the data that appears once to prevent data leakage.

The final data contains the following fields:

Canonical reaction SMILES, Catalyst1, Solvent1, Solvent2, Reagent1, Reagent2.

**Table S1**. USPTO-Condition dataset information.

| **Property** | **Information** |
| --- | --- |
| Data volume | 680741 |
| Train: validation: test | 8:1:1 |
| Categories of catalyst1 | 54 (include “null” label) |
| Categories of solvent1 | 85 (include “null” label) |
| Categories of solvent2 | 41 (include “null” label) |
| Categories of reagent1 | 223 (include “null” label) |
| Categories of reagent2 | 95 (include “null” label) |
| Categories of solvent | 87 (include “null” label) |
| Categories of reagent | 235 (include “null” label) |
| Contains temperature information | False |
| Condition label format | SMILES (e.g. CO) |

**1.2 Reaxys-TotalSyn-Condition**

We use Reaxys to export and clean this dataset with a certain strategy. We choose chemical reactions that appear in the total synthesis literature as the starting point for cleaning. The dataset curation steps are as follows:

1. We first collected fully synthetic literature, refined them to 17,612, and extracted their DOIs.
2. The Reaxys database was queried using these DOIs to derive chemical reactions in which the yield was greater than 30%.
3. Only data for single-step reactions were selected, we then deleted entries with half-reactions and catalyst numbers greater than 1 and solvent reagent numbers greater than 2.
4. Atom map of reaction SMILES were assigned using rxnmapper, resulting in 558,337 entries of data containing reaction SMILES, reaction conditions, and yields.
5. The data items containing temperature are selected and divided into training set, test set and validation set, with a ratio of 8:1:1(144103:18013:18013). When splitting the dataset, we also adopted the method of splitting with USPTO-Condition dataset to prevent data leakage.

In this dataset, we retain Reaxys' method of describing reaction conditions (Not SMILES of compounds), so that the dataset produced will be more challenging. The final data also contains the following fields:

Canonical reaction SMILES, Catalyst1, Solvent1, Solvent2, Reagent1, Reagent2.

**Table S2**. Reaxys-TotalSyn-Condition dataset information.

| **Property** | **Information** |
| --- | --- |
| Data volume | 180,129 |
| Train: validation: test | 8:1:1 |
| Categories of catalyst1 | 67 (include “null” label) |
| Categories of solvent1 | 114 (include “null” label) |
| Categories of solvent2 | 203 (include “null” label) |
| Categories of reagent1 | 1021 (include “null” label) |
| Categories of reagent2 | 820 (include “null” label) |
| Categories of solvent | 219 (include “null” label) |
| Categories of reagent | 1064 (include “null” label) |
| Contains temperature information | True |
| Condition label format | Condition Name (e.g. dichloromethane) |

**1.3 Reaxys-TotalSyn-Condition-Sampled**

1. We first subsampled the part of the Reaxys-TotalSyn-Condition dataset that has no catalysts. The method is to keep the data of all existing catalysts and keep them accounting for 15% of the total data, and carry out stratified sampling for the partial reactions without catalysts according to the reaction category.
2. Transform the reaction conditions of the remaining data into SMILES, and only keep the data whose reaction conditions exist in USPTO-Condition.
3. Delete the data that the reaction SMILES appears in the USPTO-Condition, and the final Reaxys-TotalSyn-Condition-Sampled test set data contains 7630 reaction condition data

All dataset curation scripts of USPTO-Condition, Reaxys-TotalSyn-Condition and Reaxys-TotalSyn-Condition-Sampled are available at https://github.com/wangxr0526/Parrot.

1. **Model Details**

**2.1 Masked Reaction Center Modeling**

**
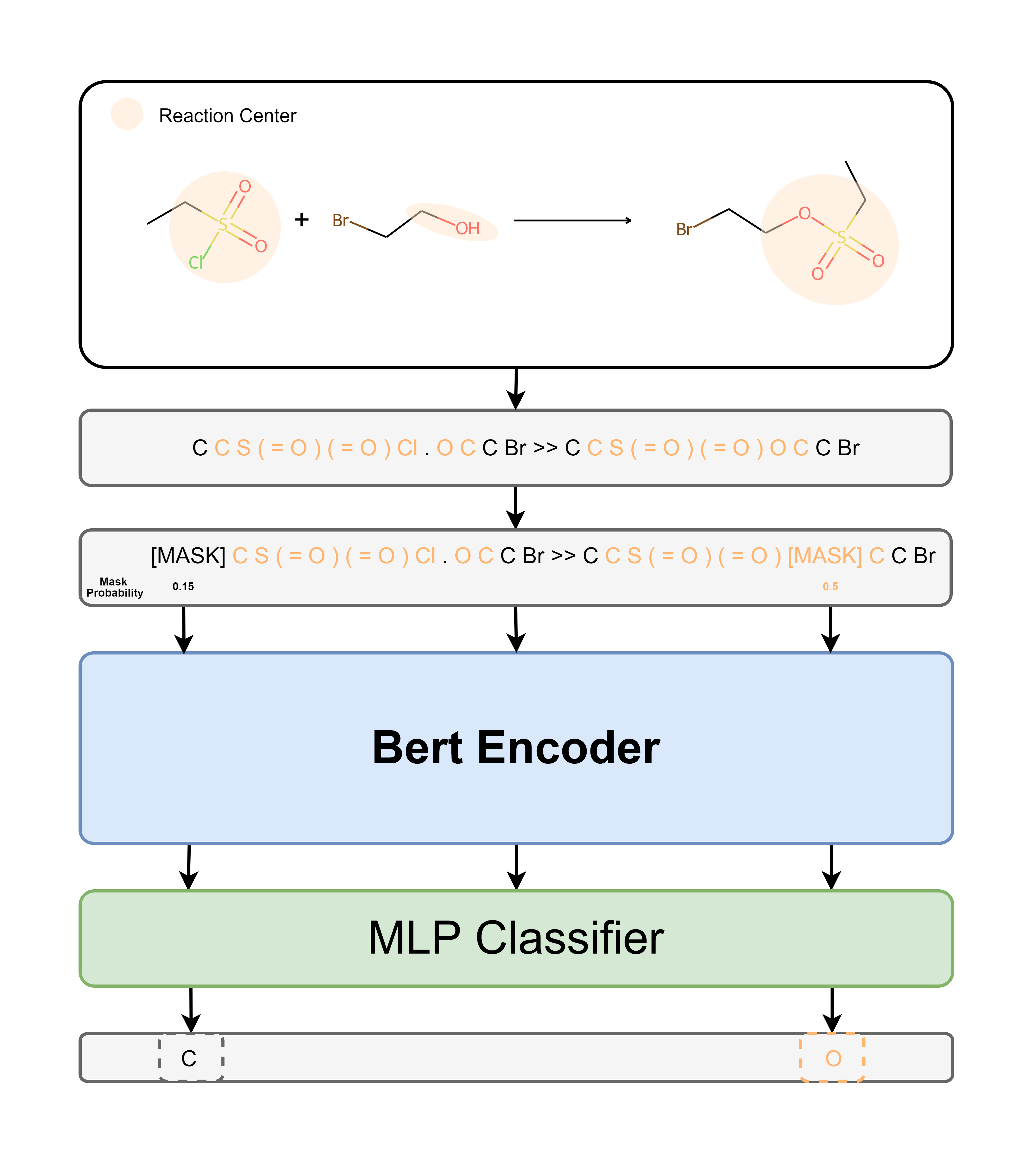
**

**Figure S1**. Schematic diagram of masked reaction center modeling.

In pretraining, Masked Reaction Center Modeling (Masked RCM) is employed to further enhance Parrot's understanding of the grammatical rules governing reaction centers. We utilize reaction templates to identify reaction centers. The extraction of these reaction templates is accomplished using rdchiral[2], and subsequently, the reaction centers of reactants and products are distinctly determined through the subgraph matching functionality of rdkit[3]. This process aids in establishing the masking probabilities of tokens within the SMILES notation. Specifically, the masking probability for tokens associated with reaction centers is set as 0.5, whereas non-reaction center tokens maintain a probability of 0.15. This facet of the pre-training strategy bears resemblance to Masked Language Modeling (Masked LM) for reactions, with the notable distinction of elevating the masking probability for reaction center tokens. This adjustment directs the model's focus towards reaction centers to a greater extent.

**2.2 Hyperparameters**

**2.2.1 Parrot**

Our code implementation is based on PyTorch, in order to simplify the implementation of the model, we adapted BertModel in simpletransformers and Transformer Decoder in PyTorch library. The hyperparameters during model training are shown in **Table S1**. We show important hyperparameters in this table. We also list the hyperparameters of Masked LM and Masked RCM in **Table S2**.

**Table S3.** Parrot model training hyperparameters. (The **bold** font indicates the optimal parameters.)

| **Model Part** | **Hyperparameter** | **Value** |
| --- | --- | --- |
| Bert Encoder | Number of Hidden Layers | 12 |
|  | Attention heads | 4 |
|  | Embedding size | 256 |
|  | Hidden size | 256 |
|  | Intermediate size | 512 |
|  | Max padding size | 512 |
| Context Condition Decoder | Number of Hidden Layers | **3**, 6 |
|  | Attention heads | **4,** 8 |
|  | Dropout rate | 0.1 |
|  | Hidden size | 256 |
|  | Embedding size | 256 |
| Temperature Decoder | Bert Encoder out size | Max padding size * 256 |
|  | Context Condition Decoder out size | 5 * 256 |
|  | Intermediate size | 512 |
|  | Loss coefficient α | 5e-4, **1e-3,** 5e-3, 1e-2 |
| Global | Max learning rate | 0.0001, **0.0002** |
|  | Enhance training learning rate | 0.00001 |
|  | Epochs | 100 |
|  | Dropout rate | 0.1 |
|  | Train batch size | 32, **64** |
|  | Warmup ratio | 0.05 |
|  | Optimizer | AdamW |

**Table S4.** Bert encoder Masked LM and Masked RCM pretraining hyperparameters.

| **Hyperparameter** | **Value** |
| --- | --- |
| Train batch size | 32 |
| Number of Hidden Layers | 12 |
| Attention heads | 4 |
| Embedding size | 256 |
| Hidden size | 256 |
| Intermediate size | 512 |
| Max padding size | 512 |
| Token masked rate | 0.15 |
| Reaction center masked rate | 0.5 (Masked RCM) |
| Epochs | 100 |
| Max learning rate | 0.0001 |
| Warmup ratio | 0.06 |
| Optimizer | AdamW |

**2.2.2 RCR**

We retrained the RCR model reported by Gao et al.[4] in both the USPTO-Condition and Reaxys-TotalSyn-Condition datasets. The data preprocessing methods for reactions, molecular fingerprint calculation details, hidden layer size of the model, and the loss function used during training were consistent with those reported in the original paper. We adjusted the batch size and learning rate during training on each dataset to obtain the optimal model weights. Throughout the training process, we saved the model checkpoint corresponding to the lowest validation set loss. After training, we used the checkpoint with the lowest loss on the validation set to calculate the final accuracy on the test set.

**Table S5.** RCR training hyperparameters. (The **bold** font indicates the optimal parameters.)

| **Hyperparameter** | **Value** |
| --- | --- |
| Train batch size | 256, **512** |
| Fingerprints size | 16384 |
| Fingerprints Hidden size | 1000 |
| Condition embedded size | 100 |
| Catalyst FFN hidden input size | 1000 |
| Solvent1 FFN hidden input size | 1100 |
| Solvent2 FFN hidden input size | 1200 |
| Reagent1 FFN hidden input size | 1300 |
| Reagent2 FFN hidden input size | 1400 |
| Temperature FFN hidden input size | 1500 |
| Condition hidden | 300 |
| Catalyst FFN output size | 54 (USPTO-Condition), 67 (Reaxys-TotalSyn-Condition) |
| Solvent1 FFN output size | 85 (USPTO-Condition), 114 (Reaxys-TotalSyn-Condition) |
| Solvent2 FFN output size | 41 (USPTO-Condition), 203 (Reaxys-TotalSyn-Condition) |
| Reagent1 FFN output size | 223 (USPTO-Condition), 1021 (Reaxys-TotalSyn-Condition) |
| Reagent2 FFN output size | 95 (USPTO-Condition), 820 (Reaxys-TotalSyn-Condition) |
| Dropout rate | 0.5 |
| Learning Rate | 0.0001, 0.0005, **0.001**, |
| Epochs | 100 |
| Optimizer | Adam |

**2.2.3 AR-GCN**

We retrained the AR-GCN model reported by Master et al.[5] in both the USPTO-Condition and Reaxys-TotalSyn-Condition datasets. We used the author's open-source code to assess the performance of AR-GCN on our curated dataset. Similar to RCR, we maintained the reported optimal parameters from the original paper, but adjusted the batch size and learning rate during training to achieve the best accuracy for our dataset. The best checkpoint selected was the one with the lowest validation set loss. It should be noted that AR-GCN can only encode chemical reactions with fewer than 3 reactants and fewer than 2 products. Therefore, during the training, validation, and testing of the model, we excluded data entries that could not be encoded by the model.

**Table S6.** AR-GCN training hyperparameters. (The **bold** font indicates the optimal parameters.)

| **Hyperparameter** | **Value** |
| --- | --- |
| Train batch size | 32, **64** |
| Hidden size | 128 |
| Epochs | 100 |
| Layers | 4 |
| Atom types | 117 |
| Dropout rate | 0.5 |
| Learning rate | 0.0001, 0.0005, **0.001**, |
| Optimizer | Adam |

**2.2.4 CIMG-Condition**

We trained five CIMG-Condition models separately to predict catalyst, solvent1, solvent2, reagent1, and reagent2. During the training of each model, we selected the best model checkpoints based on the accuracy performance on the validation set, which were then used for the final testing and evaluation of the models. We keep the parameters consistent with the original paper. The optimal parameters we used during training are listed in **Table S7**.

**Table S7.** CIMG-Condition training hyperparameters.

| **Hyperparameter** | **Value** |
| --- | --- |
| Train batch size | 500 |
| Node feature size | 10 |
| Edge feature size | 5 |
| Use reaction templates | True |
| Learning rate start | 0.001 |
| Learning rate end | 1e-07 |
| Optimizer | Adam |

**2.3 Calculation of Attention Weights**

We used the model's attention weight $a_{e}$ that connecting the atom $w$ to chemical context $C$ in the **Interpretability Analysis** section. Attention weights are computed between the encoder embedding tensors of the model and the multi-layer decoder. These decoder layers all contain multiple heads, each of which learns an attention matrix $\boldsymbol{Attention}\in\boldsymbol{R}^{N\times M}$ of weights that connect each token’s embedding $X_{i}$ in an input reaction sequence $X$ of length $N$ to every chemical context token’s embedding $Y_{j}$ in an output context condition sequence $Y$ of length $M$. Thus, each element $\boldsymbol{Attention}_{\boldsymbol{ij}}$ is the attention weight connecting $X_{i}$ to $Y_{j}$.

Each head in a multi-head attention layer will first convert the vector representation of every token $X_{i}$ or $Y_{j}$ into a key, query, and value vector using the following operations

$$\begin{aligned} K_{i}=W_{k}X_{i} Q_{j}=W_{q}Y_{j} V_{i}=W_{v}X_{i}\#\left( 1 \right) \end{aligned}$$

where $W_{k}$ , $W_{q}$, $W_{v}$ are learnable parameters. $\boldsymbol{A}_{\boldsymbol{i}}$ can be seen as the correlation probability vector of $X_{i}$ to $Y$, calculated according to the following equation:

$$\begin{aligned} \boldsymbol{A}_{\boldsymbol{i}}= Softmax\left( \frac{QK^{T}}{\sqrt{d}} \right)\boldsymbol{\#}\left( 2 \right) \end{aligned}$$

We used a similar approach to Schwaller et al.[6] to convert the attention weights of tokens to atomic attention weights. And we average the attention weight of each head as the attention weight $a$ used for analysis.

**2.4 Reaction Classification Model**

We used a neural network classifier based on rxnfp[7] to assign reaction categories to our cleaned dataset, training on the pistachio dataset[8] and achieved 97.8% accuracy on the pistachio test set. It is close to the method reported by Schwaller et al.[7]

**2.5 Attention Weights Visualization**


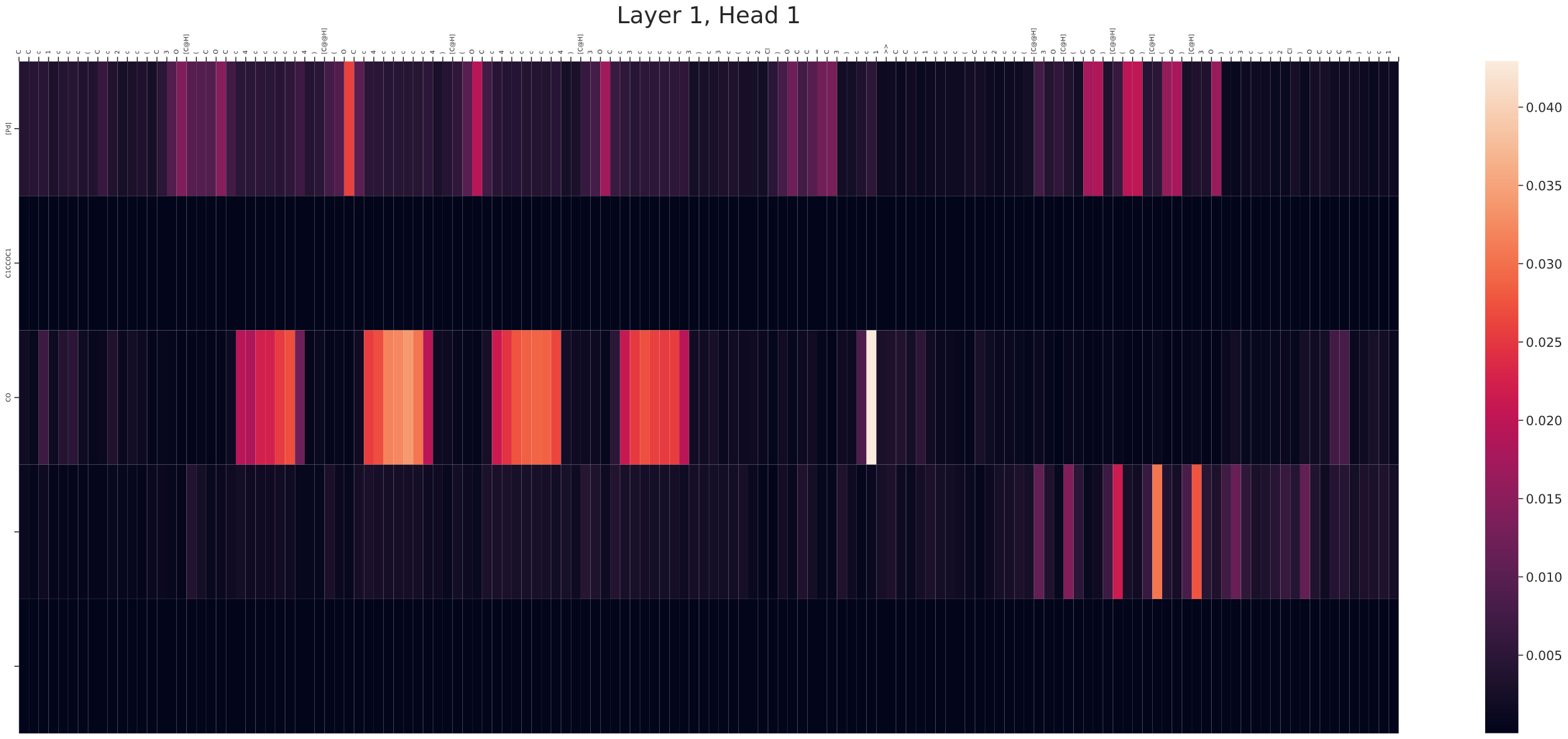


**Figure S2.** High-resolution attention map of Figure 5B (Layer 1, Head 1).


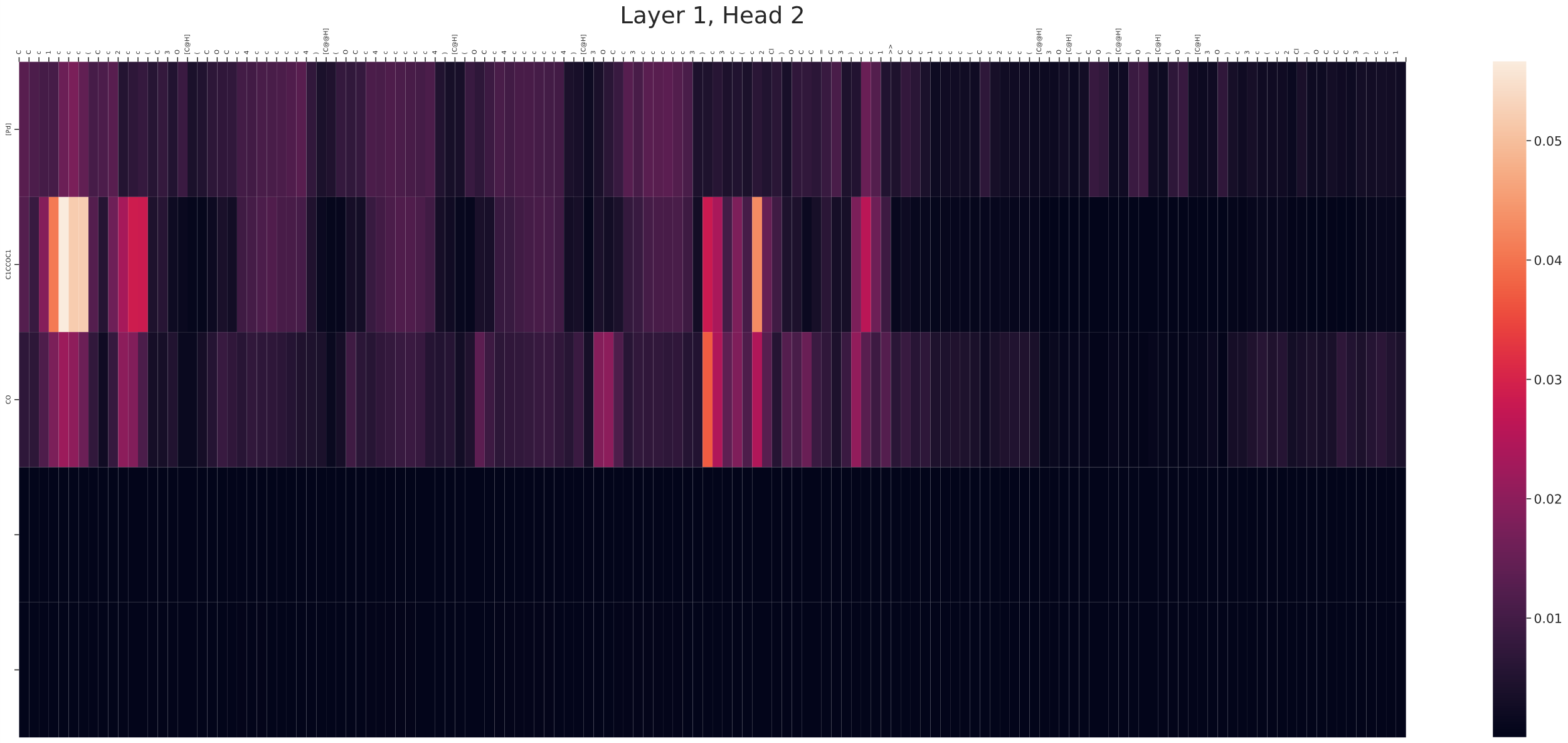


**Figure S3.** High-resolution attention map of Figure 5B (Layer 1, Head 2).


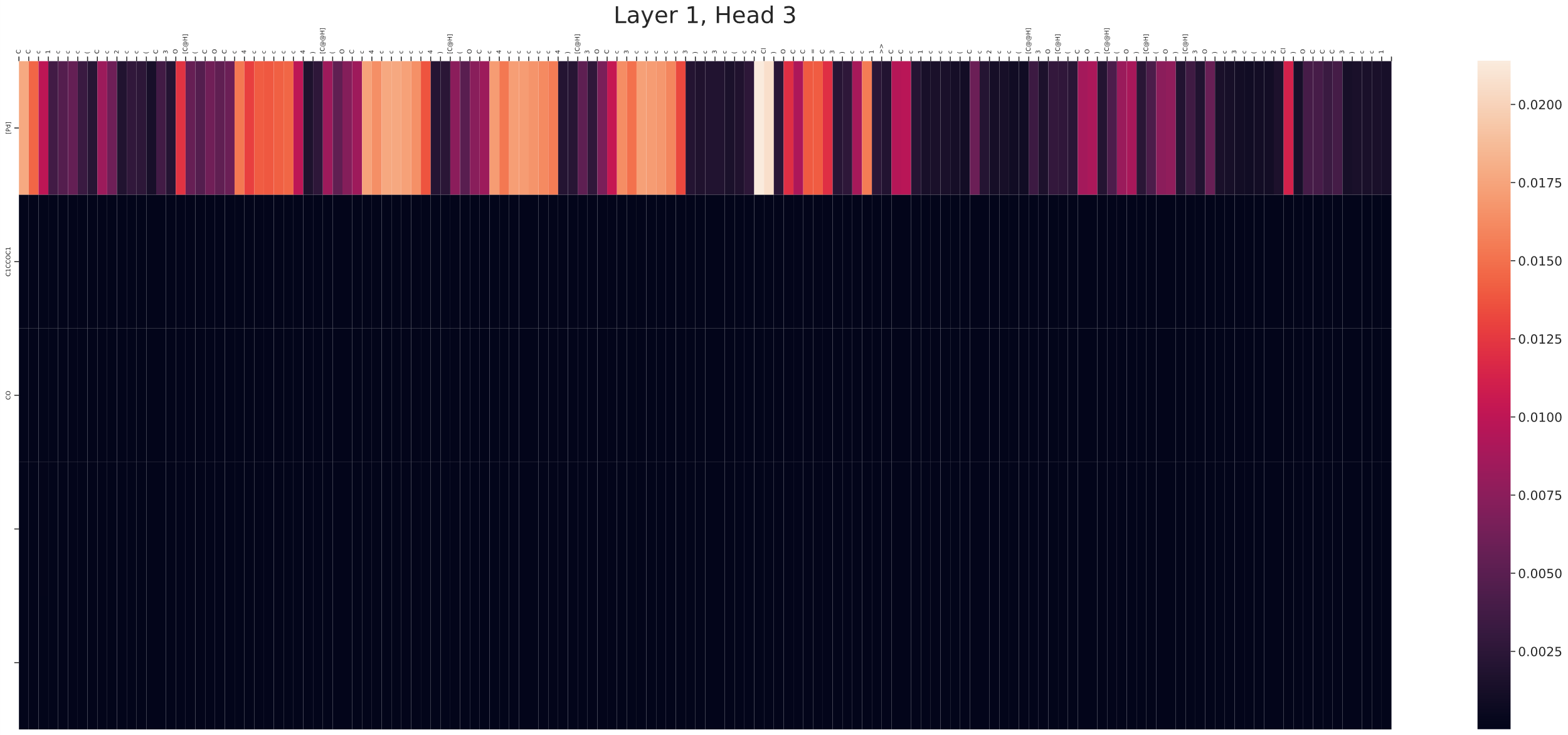


**Figure S4.** High-resolution attention map of Figure 5B (Layer 1, Head 3).


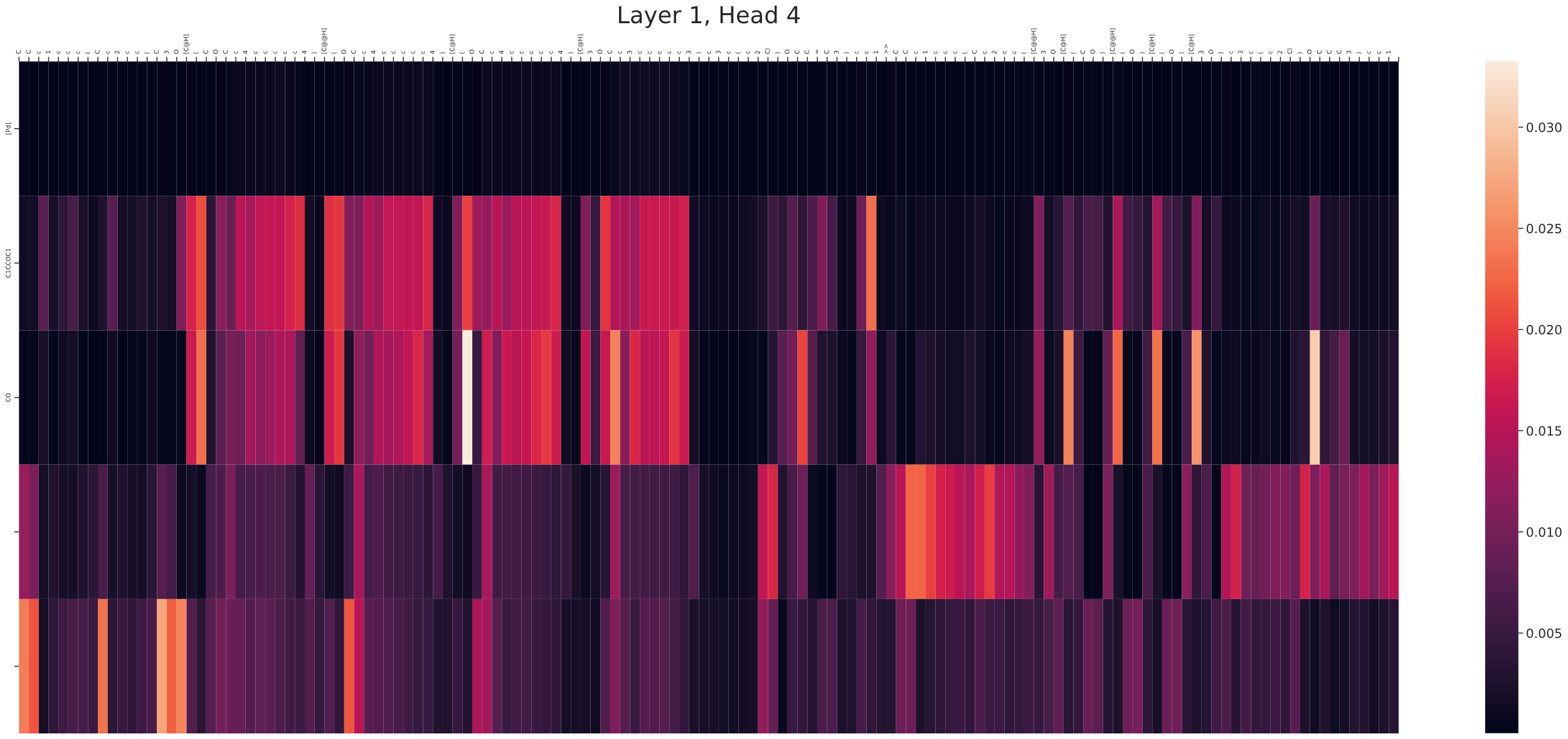


**Figure S5.** High-resolution attention map of Figure 5B (Layer 1, Head 4).


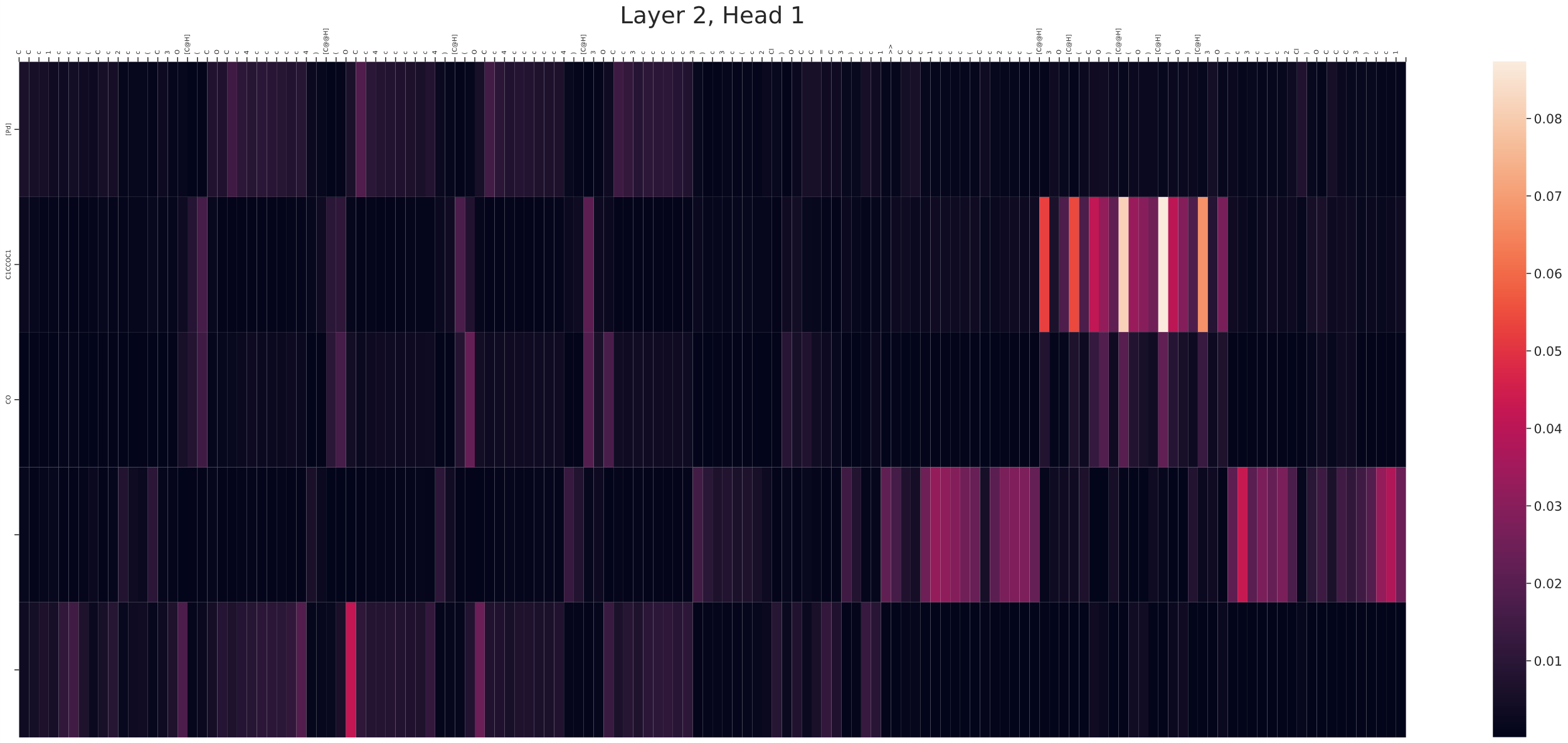


**Figure S6.** High-resolution attention map of Figure 5B (Layer 2, Head 1).


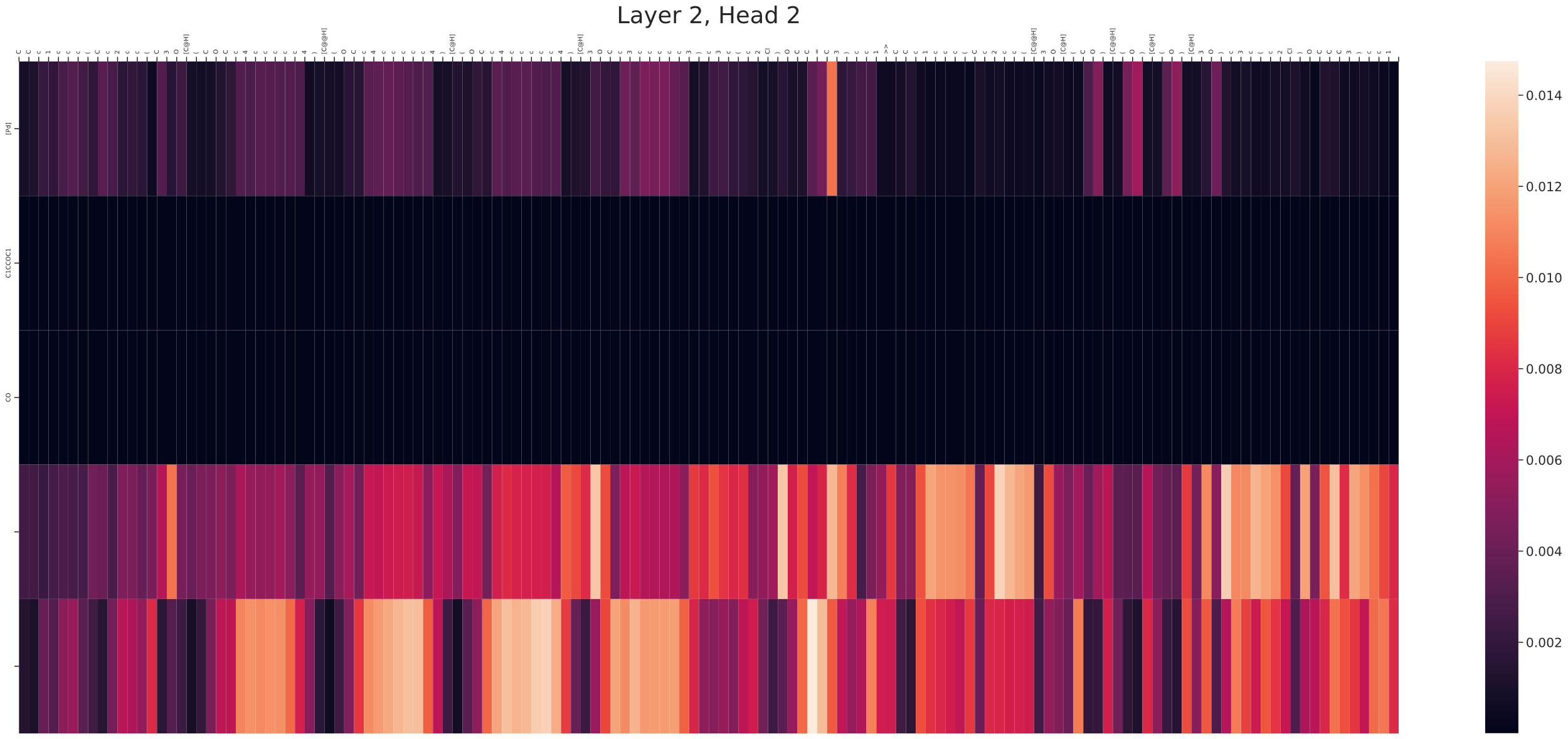


**Figure S7.** High-resolution attention map of Figure 5B (Layer 2, Head 2).


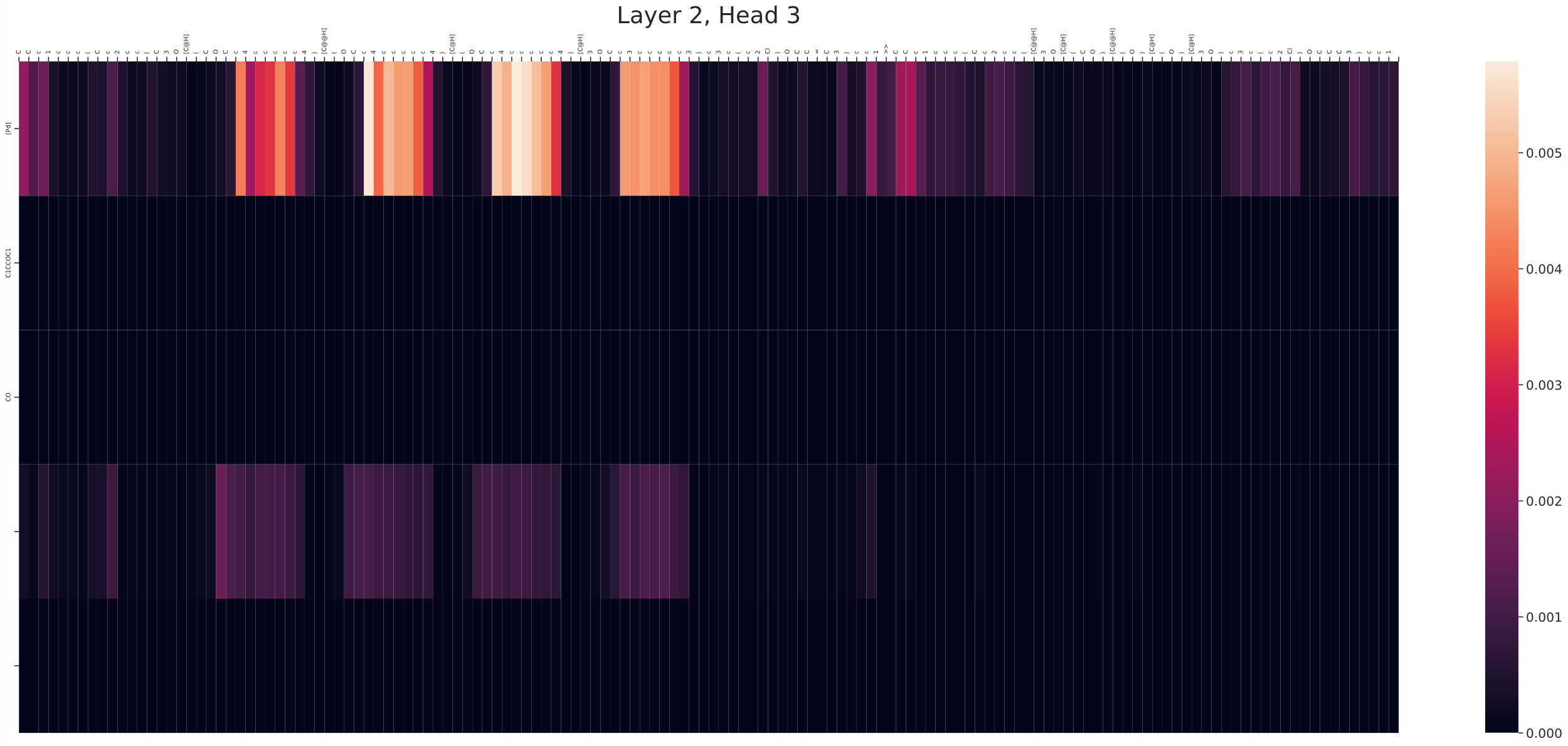


**Figure S8.** High-resolution attention map of Figure 5B (Layer 2, Head 3).


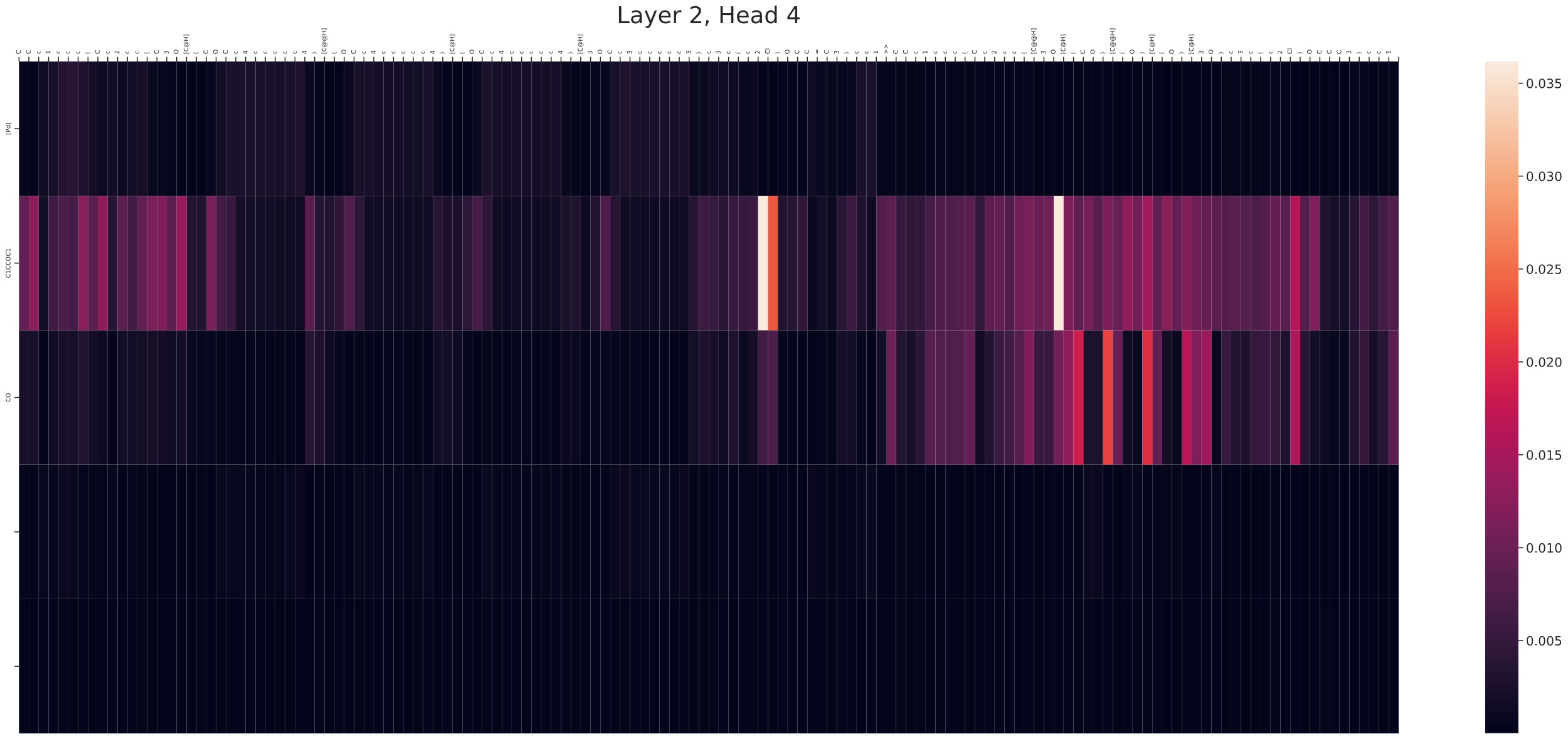


**Figure S9.** High-resolution attention map of Figure 5B (Layer 2, Head 4).


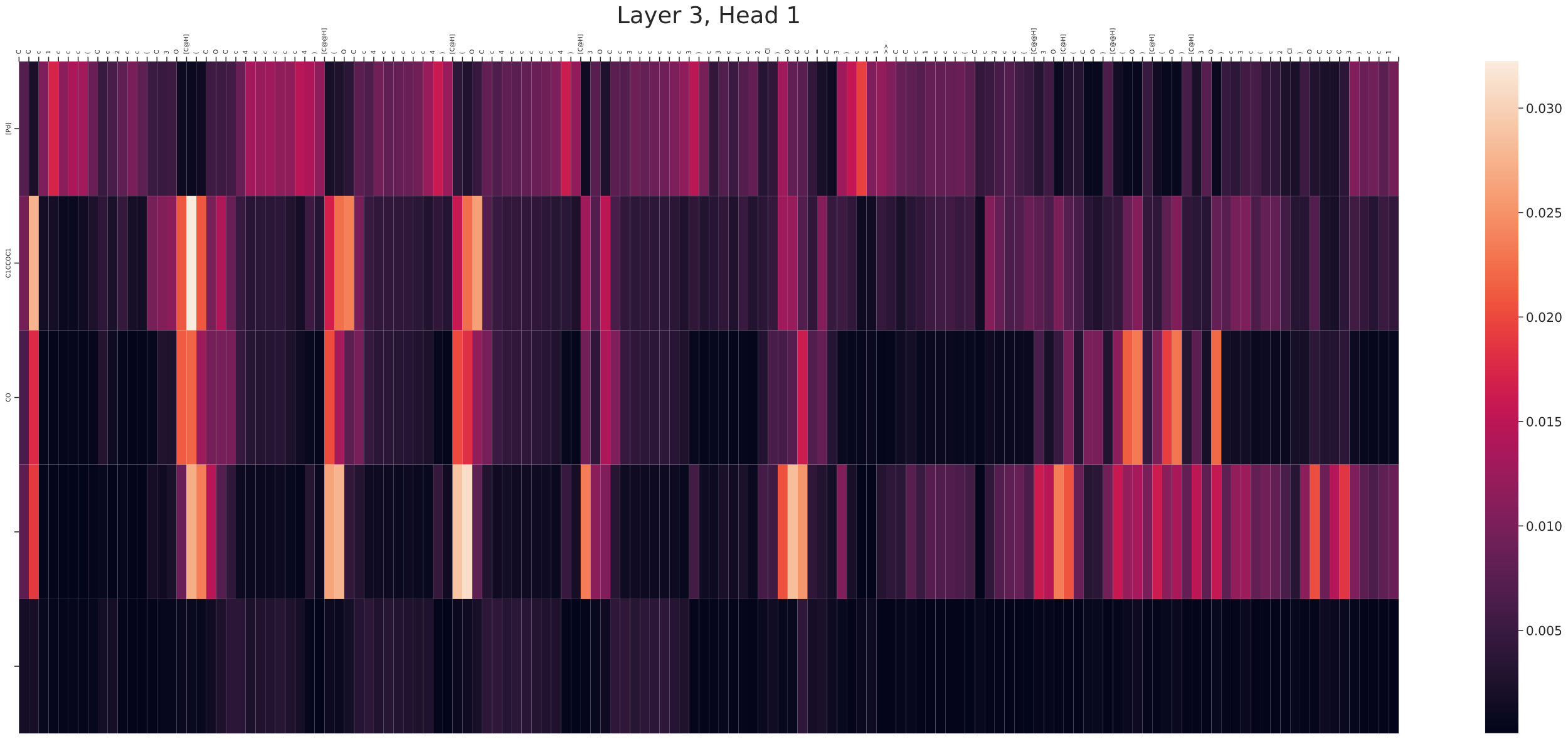


**Figure S10.** High-resolution attention map of Figure 5B (Layer 3, Head 1).


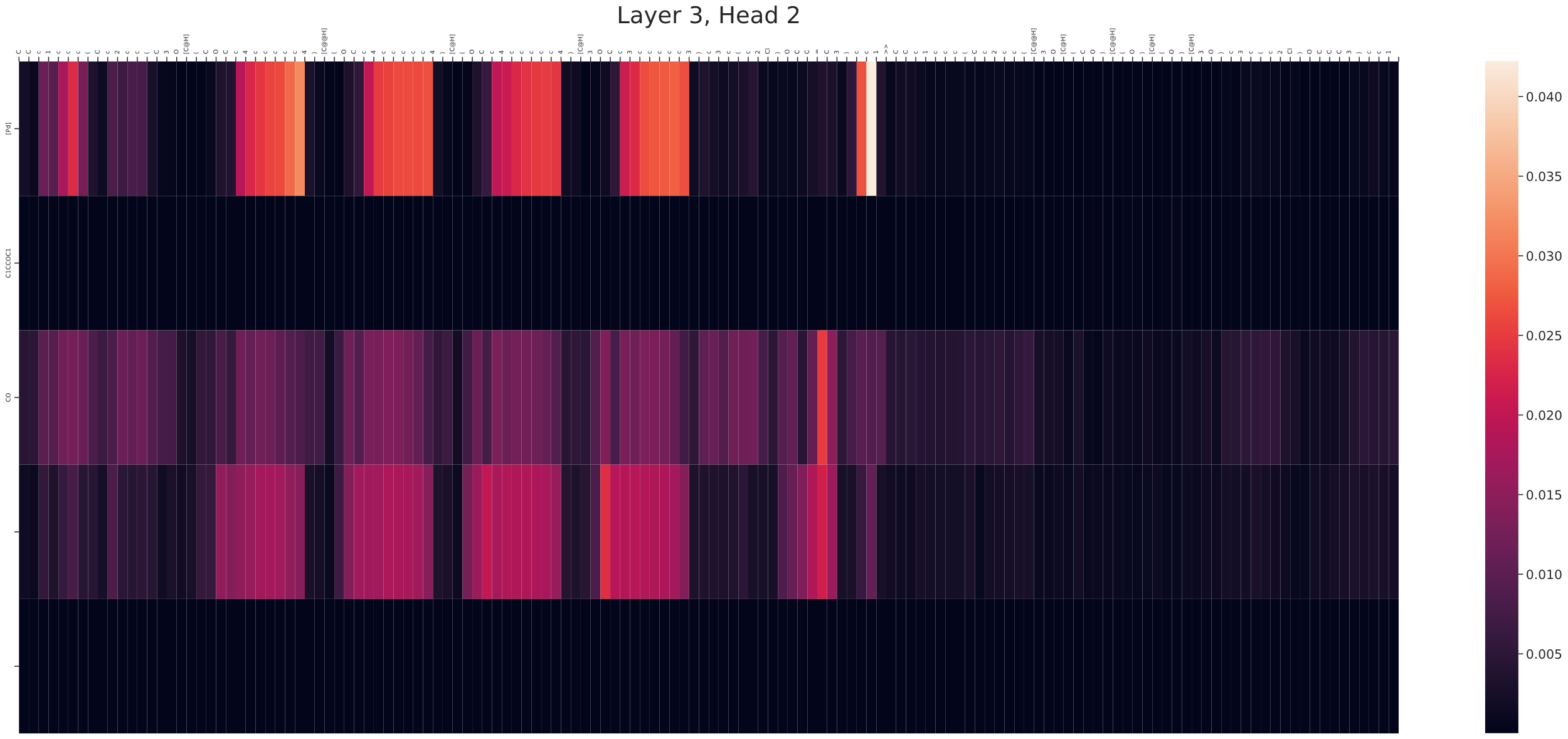


**Figure S11.** High-resolution attention map of Figure 5B (Layer 3, Head 2).


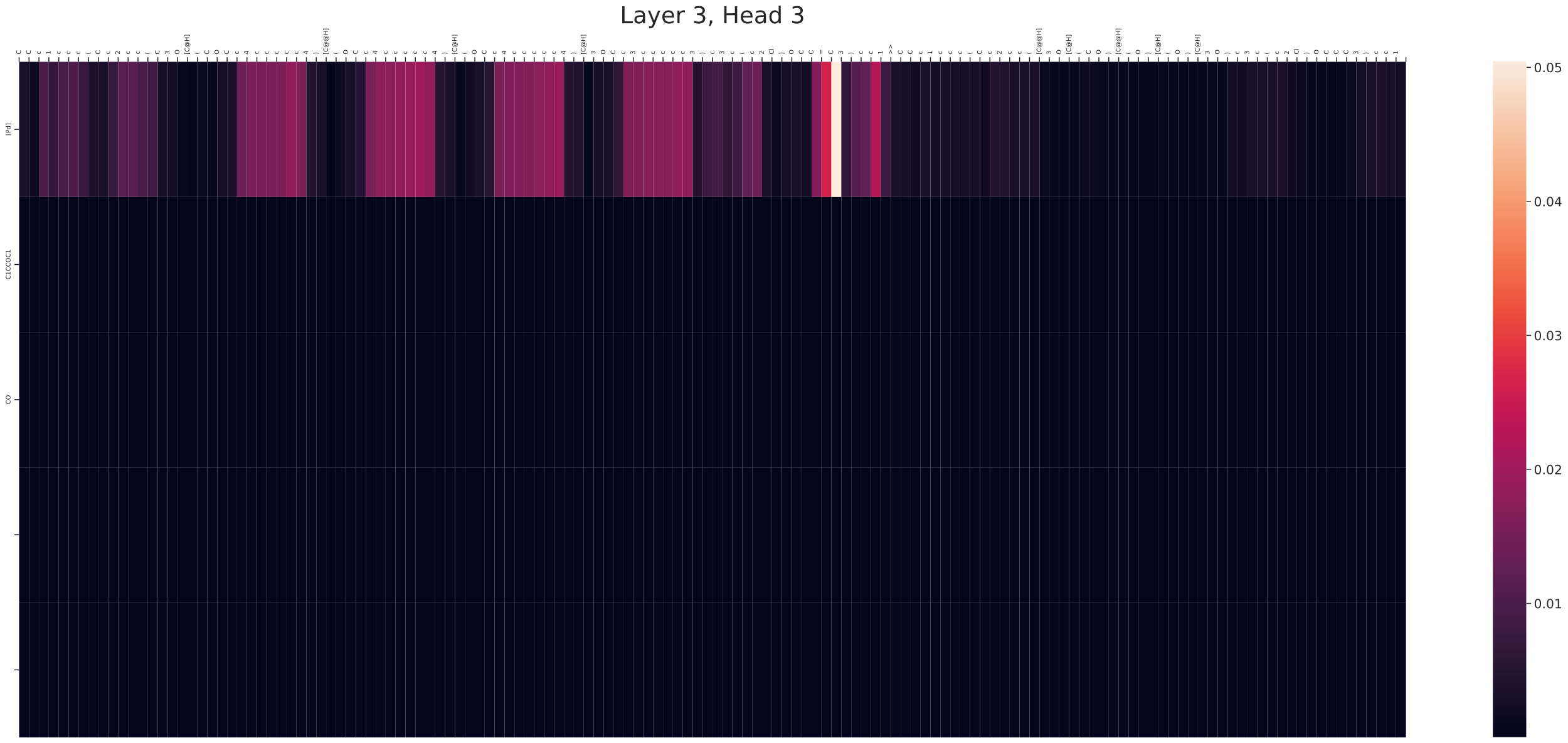


**Figure S12.** High-resolution attention map of Figure 5B (Layer 3, Head 3).


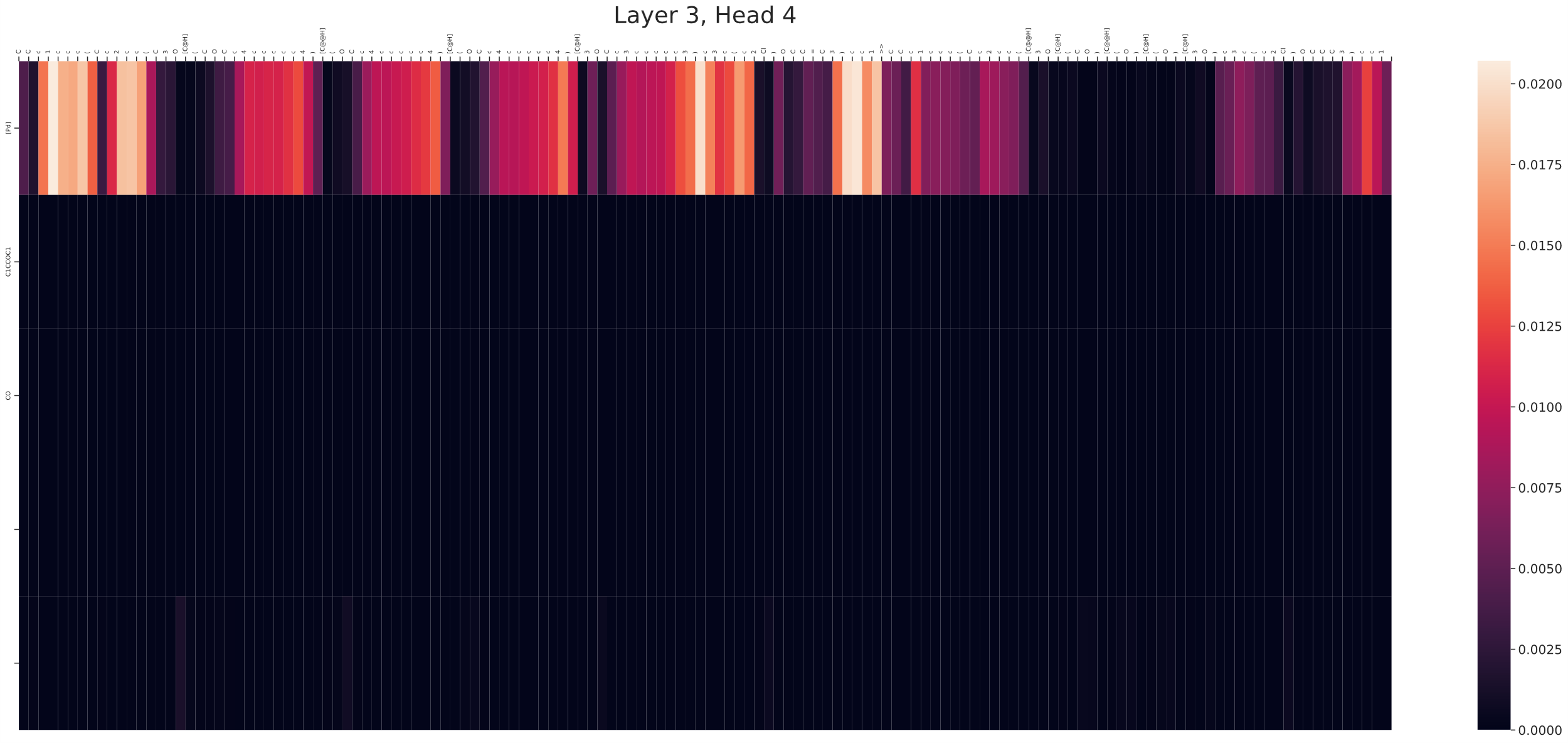


**Figure S13.** High-resolution attention map of Figure 5B (Layer 3, Head 4).

**2.6 Parameter for Analysis of the Parrot's Understanding of Reaction Centers**

**Table S8.** Parameter optimization space and the optimal parameters for Analysis of the Parrot's Understanding of Reaction Centers.

| **Evaluation Parameter** | **Values** | **Best value** |
| --- | --- | --- |
| $k$ | {-0.5, -0.4, -0.3, -0.2, -0.1, 0.0, 0.1, 0.2, 0.3, 0.4, 0.5} | -0.2 |
| $\mathrm{ConditionType}$ | {c, s1, s2, r1, r2} | c |
| $Head (cross attention)$ | {1,2,3,4} | 4 |
| $Layer (cross attention)$ | {1,2,3} | 2 |
| $\mathrm{Head}(self attention)$ | {1,2,3,4} | 4 |
| $\mathrm{Layer}(self attention)$ | {1,2,3,…,11,12} | 5 |
| n | {1,2,3,4,5,6} | 2 |

1. **Classification of Solvents and Reagents**

**3.1 Solvents Classification by Solvent Similarity Index**[9]

We classified solvents according to the dendrogram of 14 similarity regions reported in the paper supporting information (<https://www.rsc.org/suppdata/d0/cp/d0cp01570a/d0cp01570a1.pdf>) by Driver et al. [9] If the solvent was not included in the solvents studied in this work, it was classified into a separate class.

**3.2 Reagents Classification by key structure fingerprints**

We defined some substructures that are very related to the properties of the reagents, and used the unique hot vector with or without the occurrence of substructures as the key substructure fingerprint, and those with the same fingerprint were defined as the same class of reagents. The key substructures are defined according to the **Table S9**. Note that reagents that do not contain any of the substructures we defined are counted as a separate class.

**Table S9.** Feature definition of key substructure fingerprint.

| **Smarts** | **Represented substructure** |
| --- | --- |
| [!H0;#7,#8,#9] | Hydrogen bond donor |
| [!$([#6,H0,-,-2,-3])] | Hydrogen bond donor |
| [!$([#6,F,Cl,Br,I,o,s,nX3,#7v5,#15v5,#16v4,#16v6,*+1,*+2,*+3])] | Hydrogen-bonded receptors |
| [#6,#7;R0]=[#8] | Hydrogen-bonded receptors |
| [CX3](=[OX1])[F,Cl,Br,I] | Acyl halide compounds |
| [$([#16X4](=[OX1])(=[OX1])([#6])[OX2H,OX1H0-]),$([#16X4+2]([OX1-])([OX1-])([#6])[OX2H,OX1H0-])] | Sulfonic acid |
| [$(P(=[OX1])([$([OX2H]),$([OX1-]),$([OX2]P)])([$([OX2H]),$([OX1-]),$([OX2]P)])[$([OX2H]),$([OX1-]),$([OX2]P)]),$([P+]([OX1-])([$([OX2H]),$([OX1-]),$([OX2]P)])([$([OX2H]),$([OX1-]),$([OX2]P)])[$([OX2H]),$([OX1-]),$([OX2]P)])] | Phosphoric acid |
| [$([OH]-*=[!#6])] | Hydroxyl acidic |
| [CX3](=O)[OX2H1] | Carboxylic acid |
| [$([CX3]=[OX1]),$([CX3+]-[OX1-])] | Carbonyl group |
| [CX3](=[OX1])C | Carbonyl with Carbon |
| [OX1]=CN | Carbonyl with Nitrogen. |
| [CX3](=[OX1])O | Carbonyl with Oxygen |
| [CX3H1](=O)[#6] | Aldehyde |
| [CX3](=[OX1])[OX2][CX3](=[OX1]) | Anhydride |
| [NX3][CX3](=[OX1])[#6] | Amide |
| Continues on the next page | |
|  | |

**Table S9** **(Continued).**

| **Smarts** | **Represented substructure** |
| --- | --- |
| [CX3](=O)[OX1H0-,OX2H1] | Carboxylic acid or conjugate base |
| [NX3][CX3]=[NX3+] | Amidinium |
| [NX3,NX4+][CX3](=[OX1])[OX2,OX1-] | Carbamate |
| [CX3](=O)[O-] | Carboxylate Ion |
| [CX3](=[OX1])(O)O | Carbonic Acid or Carbonic Ester |
| [NX2-] | Anionic divalent Nitrogen |
| [OX2H+]=* | Oxenium Oxygen |
| [OX3H2+] | Oxonium Oxygen |
| [#6+] | Carbocation |
| [$([cX2+](:*):*)] | sp2 cationic carbon |
| [$([NX1-]=[NX2+]=[NX1-]),$([NX1]#[NX2+]-[NX1-2])] | Azide ion |
| [+1]~*~*~[-1] | +1 charged atom separated by any 3 bonds from a -1 charged atom |
| [+] | cation |
| [-] | anion |
| [$([cX3](:*):*),$([cX2+](:*):*)] | Aromatic sp2 carbon |
| [$([cX3](:*):*),$([cX2+](:*):*),$([CX3]=*),$([CX2+]=*)] | Any sp2 carbon |
| [C] | Contains carbon |
| [N] | Contains nitrogen |
| [O] | Contains oxygen |
| [P] | Contains phosphorus |
| [c] | Contains aromatic carbon |
| [n] | Contains aromatic nitrogen |
| [S] | Contains sulfur |
| [B] | Contains boron |
| [Na,K,Li] | Contains common alkali metal elements |
| [Si] | Contains silicon |

The classification code can be viewed at:

https://github.com/wangxr0526/Parrot/blob/master/preprocess_script/uspto_script/condition_classfication.ipynb.

1. **Extended Results**

**Table S10.** Results of the AR-GCN model on the USPTO-Condition test set

| Models | Chemical Context Condition Accuracy↑ | | | | | | |
| --- | --- | --- | --- | --- | --- | --- | --- |
|  | Conditions | Top-1 | Top-3 | Top-5 | Top-10 | Top-15 |  |
| AR-GCN^c^[5] | c | 0.9024 | 0.9024 | 0.9024 | 0.9024 | 0.9024 |  |
|  | s1 | 0.4114 | 0.5787 | 0.6295 | 0.6635 | 0.6650 |  |
|  | s2 | 0.8093 | 0.8093 | 0.8093 | 0.8093 | 0.8093 |  |
|  | r1 | 0.4200 | 0.5740 | 0.6667 | 0.7515 | 0.7622 |  |
|  | r2 | 0.7486 | 0.7486 | 0.7486 | 0.7486 | 0.7486 |  |
|  | overall**^b^** | 0.1460 | 0.2374 | 0.2733 | 0.3121 | 0.3261 |  |
| AR-GCN-Corr^d^ [5] | c | 0.8082 | 0.8082 | 0.8082 | 0.8082 | 0.8082 |  |
|  | s1 | 0.3684 | 0.5183 | 0.5638 | 0.5942 | 0.5955 |  |
|  | s2 | 0.7248 | 0.7248 | 0.7248 | 0.7248 | 0.7248 |  |
|  | r1 | 0.3761 | 0.5140 | 0.5971 | 0.6730 | 0.6826 |  |
|  | r2 | 0.6704 | 0.6704 | 0.6704 | 0.6704 | 0.6704 |  |
|  | overall | 0.1308 | 0.2126 | 0.2448 | 0.2795 | 0.2920 |  |
| CIMG-Condition[10] | c | 0.9146 | 0.9146 | 0.9146 | 0.9146 | 0.9146 |  |
|  | s1 | 0.4218 | 0.6139 | 0.6542 | 0.6780 | 0.6789 |  |
|  | s2 | 0.8110 | 0.8110 | 0.8110 | 0.8110 | 0.8110 |  |
|  | r1 | 0.4351 | 0.5685 | 0.6665 | 0.7462 | 0.7598 |  |
|  | r2 | 0.7574 | 0.7574 | 0.7574 | 0.7574 | 0.7574 |  |
|  | overall | 0.1839 | 0.2714 | 0.3026 | 0.3391 | 0.3525 |  |

**^a^**c, s1, s2, r1, and r2 refer to catalyst, solvent 1, solvent 2, reagent 1, and reagent 2, respectively.

^b^overall: c, s1, s2, r1, r2.

^c^We only calculated the accuracy based on the data that the model could encode. In this case, the testing included a total of 60,965 data entries, excluding the 7,110 entries that could not be encoded. The accuracy was computed solely on the encoded data.

^d^The data that the model could not encode were considered as prediction errors. In this case, the testing included a total of 68,075 data entries, including those that could not be encoded by the model.

**Table S11.** Results of the AR-GCN model on the Reaxys-TotalSyn-Condition test set^a^

| Models | Chemical Context Condition Accuracy↑ | | | | | |
| --- | --- | --- | --- | --- | --- | --- |
|  | Conditions | Top-1 | Top-3 | Top-5 | Top-10 | Top-15 |
| **Alpha**: Test results for the portion of the test set without catalyst^b^ | | | | | | |
| RelGCN^d^ | s1 | 0.4790 | 0.6303 | 0.6775 | 0.7235 | 0.7293 |
|  | r1 | 0.3740 | 0.4971 | 0.5606 | 0.6078 | 0.6123 |
|  | s1r1 | 0.2407 | 0.3761 | 0.4301 | 0.4830 | 0.5067 |
| RelGCN-Corr^e^ | s1 | 0.4267 | 0.5615 | 0.6036 | 0.6445 | 0.6497 |
|  | r1 | 0.3332 | 0.4428 | 0.4994 | 0.5415 | 0.5455 |
|  | s1r1 | 0.2144 | 0.3351 | 0.3832 | 0.4303 | 0.4514 |
| CIMG-Condition[10] | s1 | 0.5307 | 0.7123 | 0.7408 | 0.7614 | 0.7635 |
|  | r1 | 0.4204 | 0.5183 | 0.5741 | 0.6237 | 0.6287 |
|  | s1r1 | 0.3010 | 0.4202 | 0.4695 | 0.5199 | 0.5379 |
| **Beta**: Test results for the portion of the test set containing the catalyst^c^ | | | | | | |
| RelGCN^f^ | c1 | 0.0163 | 0.0679 | 0.1168 | 0.4837 | 0.4837 |
|  | s1 | 0.2609 | 0.4022 | 0.4755 | 0.5163 | 0.5163 |
|  | r1 | 0.2255 | 0.3342 | 0.3940 | 0.4130 | 0.4130 |
|  | c1s1r1 | 0.0109 | 0.0326 | 0.0543 | 0.0897 | 0.1413 |
| RelGCN-Corr^g^ | c1 | 0.0149 | 0.0622 | 0.1069 | 0.4428 | 0.4428 |
|  | s1 | 0.2388 | 0.3682 | 0.4353 | 0.4726 | 0.4726 |
|  | r1 | 0.2064 | 0.3059 | 0.3607 | 0.3781 | 0.3781 |
|  | c1s1r1 | 0.0100 | 0.0298 | 0.0497 | 0.0821 | 0.1293 |
| CIMG-Condition[10] | c1 | 0.1072 | 0.1072 | 0.1072 | 0.1072 | 0.1072 |
|  | s1 | 0.3242 | 0.4963 | 0.5212 | 0.5312 | 0.5312 |
|  | r1 | 0.3791 | 0.4738 | 0.5636 | 0.6334 | 0.6409 |
|  | c1s1r1 | 0.0673 | 0.0848 | 0.0898 | 0.0923 | 0.0923 |

**^a^**c, s1, s2, r1, and r2 refer to catalyst, solvent 1, solvent 2, reagent 1, and reagent 2, respectively.

^b^Portfolio of predicted results: s1 top3, r1 top5.

^c^Portfolio of predicted results: c1 top2, s1 top3, r1 top5.

^d^We only calculated the accuracy based on the data that the model could encode. In this case, the testing included a total of 15,689 data entries, excluding the 1,922 entries that could not be encoded. The accuracy was computed solely on the encoded data.

^e^The data that the model could not encode were considered as prediction errors. In this case, the testing included a total of 17,661 data entries, including those that could not be encoded by the model.

^f^We only calculated the accuracy based on the data that the model could encode. In this case, the testing included a total of 368 data entries, excluding the 34 entries that could not be encoded. The accuracy was computed solely on the encoded data.

^g^The data that the model could not encode were considered as prediction errors. In this case, the testing included a total of 402 data entries, including those that could not be encoded by the model.

1. **Web GUI**

We include a web application developed based on the flask framework in the open-source code to facilitate researchers to use Parrot to predict reaction conditions. After starting the web application, the interface shown in **Figure S14** will be displayed. The GUI supports three input methods: draw reaction, paste reaction SMILES and upload .txt file with reaction SMILES, and the final output is presented and exported in tabular form.


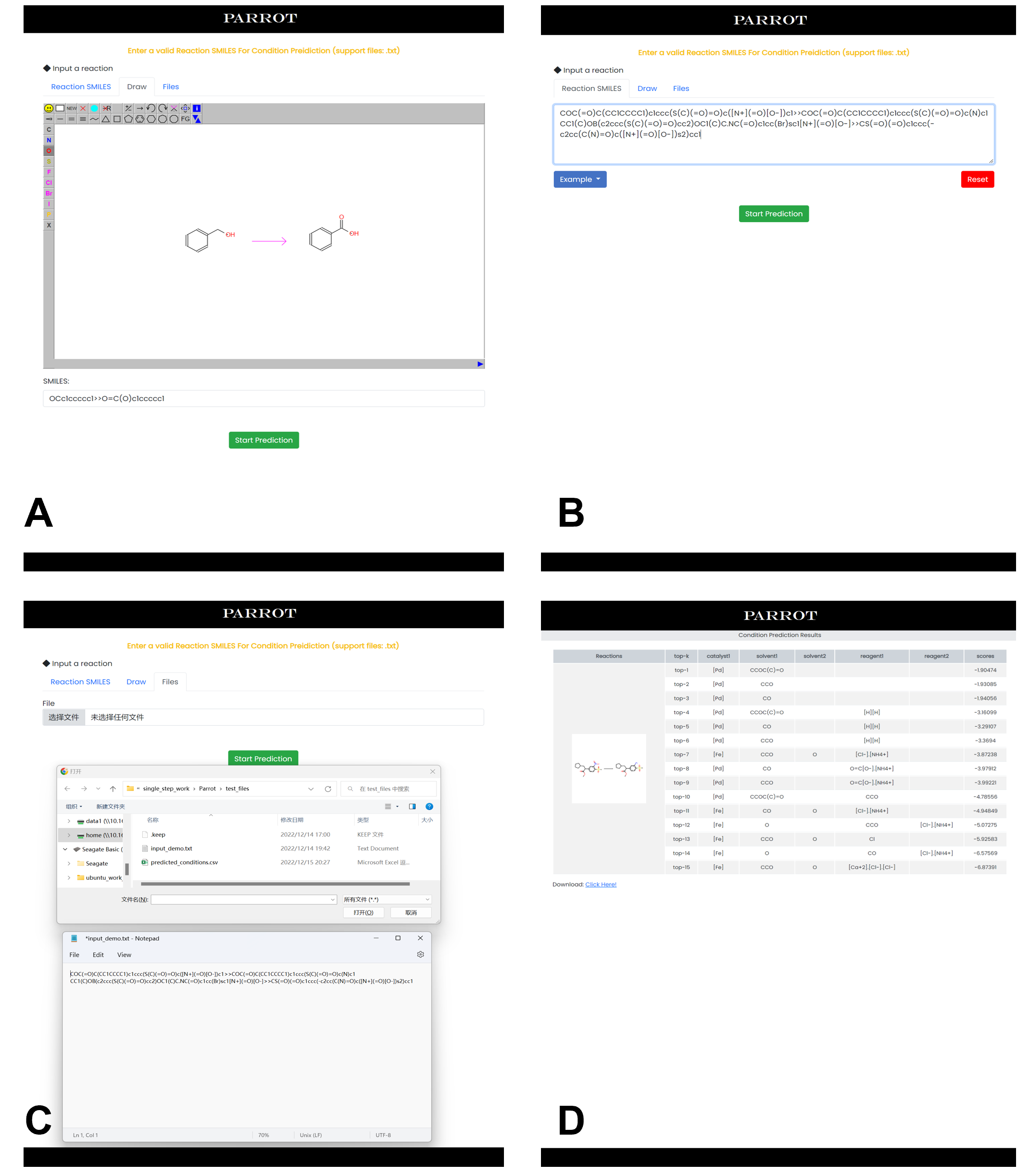


**Figure S14.** Web GUI of Parrot.

1. **Reaction Similarity Calculation**

In the **Generalizable Prediction Capabilities Across Reaction Space** Section, we utilized reaction difference fingerprints and Tanimoto similarity[11] to assess the differences between the Reaxys-TotalSyn-Condition-Sampled test set and the USPTO-Condition dataset. The specific methodology is described as follows:

- - - 1. For each reaction sample in a dataset containing n samples, the ***CreateDifferenceFingerprintForReaction*** function from RDKit was used to compute the difference fingerprint of the reaction.
      2. For each reaction sample, the similarity with all reactions in the compared dataset (containing m samples) was measured using Tanimoto similarity as the similarity metric. The calculation formula is as follows:

$$Tanimoto\left( \mathbf{x},\mathbf{y} \right)=\frac{\sum x_{i}y_{i}}{\sum x_{i}^{2}+\sum y_{i}^{2}-\sum x_{i}y_{i}}$$

Where x and y represent the reaction difference fingerprints vectors that describe the reactions. After this step, we obtain n similarity vectors of length m.

3. The average similarity was calculated by taking the mean of the similarity values of the top-5 most similar samples in the similarity vector of each reaction sample. This provided a measure of the sample's similarity to the compared dataset.

4. The similarity distribution of the two datasets was analyzed to visually demonstrate the similarity between the datasets.

Reaction difference fingerprints can describe structural changes between reactants and products in a reaction, capturing key reaction features. By comparing the reaction difference fingerprints of different reactions, one can evaluate the similarity or dissimilarity between them. If two reactions have similar reaction difference fingerprints, it indicates that their changes are alike, possibly involving similar transformation steps or even similar reaction conditions. On the other hand, if there is a significant difference in the difference fingerprints, it suggests notable structural variations between the two reactions, with potentially significant differences in the required reaction conditions.

Through the analysis of reaction similarity based on reaction difference fingerprints and tanimoto similarity, we can clearly observe the reaction similarity between the training set and the test set. This allows us to compare the variations in predictive performance of reaction conditions among different models when the gap between the training set and the test set widens.

1. **Top-k Accuracy Calculation**


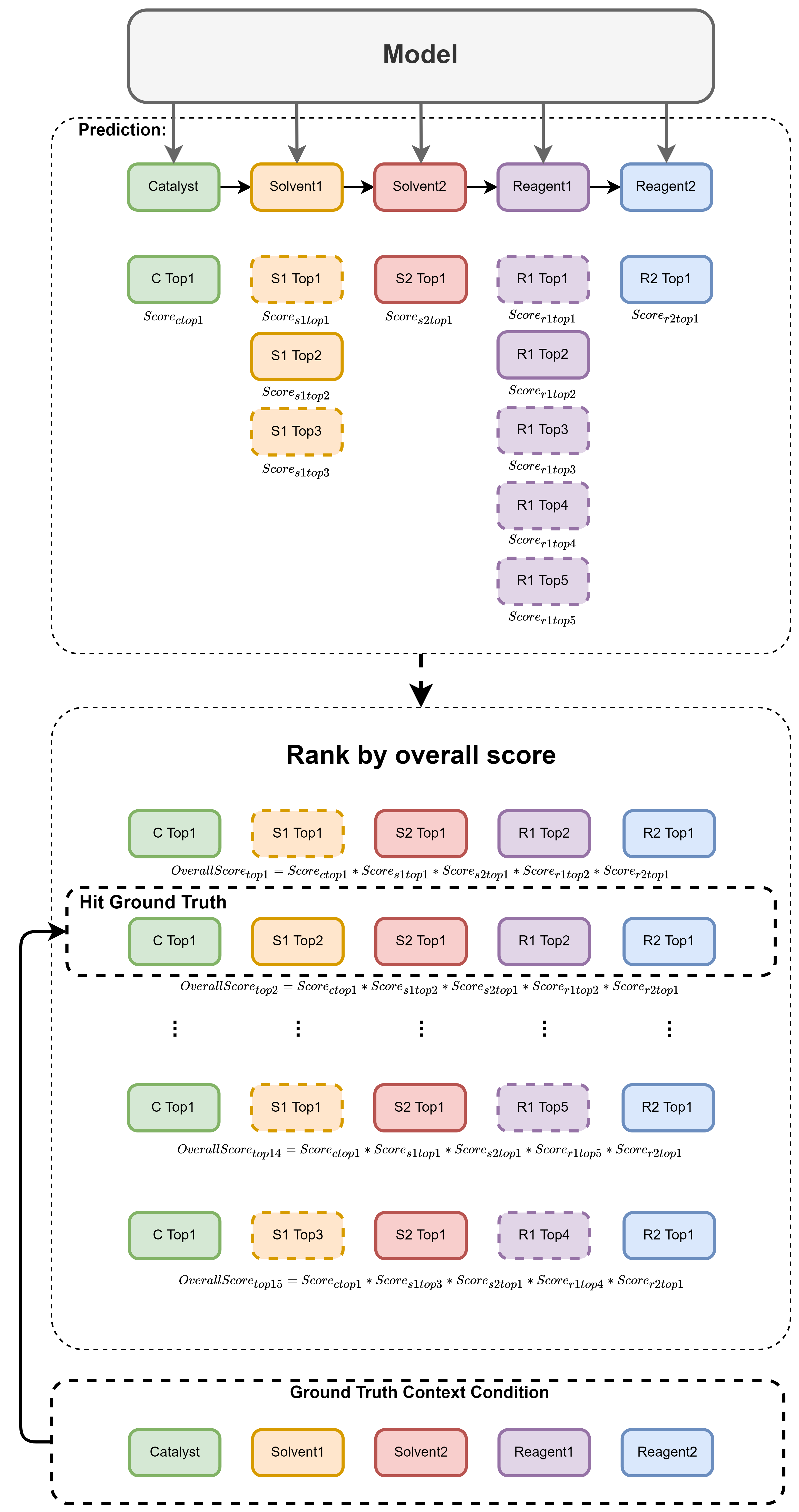


**Figure S15.** Top-k accuracy calculation method diagram. In this example, the prediction ranked second overall correctly identified the reaction conditions, while the top-ranked prediction did not. Therefore, when calculating the top-k accuracy, the top-1 accuracy is 0, and the accuracy from top-2 to top-15 is 1.

In the main text, we utilized Top-k accuracy to evaluate the performance of reaction condition prediction tasks. The number of candidate selections for each reaction condition category is determined by the sparsity level of the corresponding condition labels. Models predict fewer candidate selections (smaller top-k values) for sparser reaction condition categories and more candidate selections (larger top-k values) for denser reaction condition categories. The calculation process is illustrated in **Figure S15**. The models sequentially predict each category of reaction conditions, outputting the top-k predicted results and their corresponding softmax probability scores. These results are combined for each category of reaction conditions, and the final prediction is determined by sorting the combined results based on the product of softmax probability scores for each prediction. It is worth noting that CIMG requires the establishment of five models to complete the prediction of all reaction condition categories.

**8. Variations in Predictive Performance across Reaction Space**


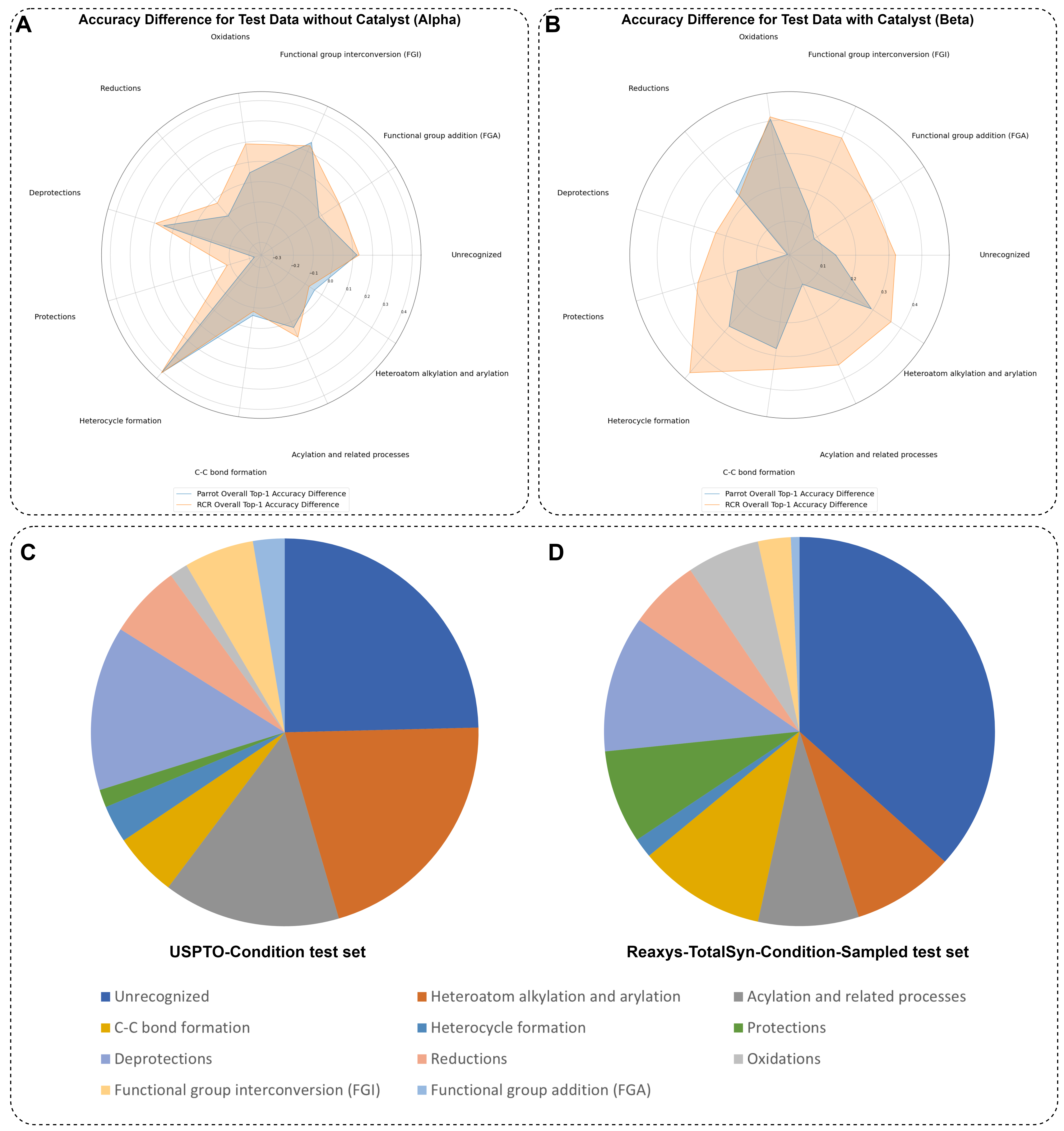


**Figure S16.** Illustration of overall top-1 accuracy variations for different reaction types across reaction space. The blue radar chart represents the decrease values in Top-1 accuracy of the Parrot model when transitioning from the USPTO-Condition test set to the Reaxys-TotalSyn-Condition-Sampled test set. The orange radar chart represents the decrease values in Top-1 accuracy of the RCR model during the same transition. **Figure A** displays the results of the test data without catalyst. In this case, the term "Overall Top-1 Accuracy" refers to the accuracy when both solvent1 and reagent1 are predicted correctly. **Figure B** shows the results of the test data with catalyst. Here, "Overall Top-1 Accuracy" represents the accuracy when catalyst, solvent1 and reagent1 are all predicted correctly. **Figure C** illustrates the distribution of the USPTO-Condition test set, while **Figure D** illustrates the distribution of the Reaxys-TotalSyn-Condition-Sampled test set. The reaction type of "Resolutions" was not taken into consideration during the creation of Reaxys-TotalSyn-Condition-Sampled due to its limited occurrence.

From the **Figure S16**, we can observe that the decrease in accuracy for most reaction types in Parrot's prediction across reaction space is significantly smaller compared to RCR's prediction results. We found that in the subset without catalyst, except for the same accuracy change of Parrot and RCR models for two reaction types: "Functional group interconversion " and "Heterocycle formation ", Parrot shows much less accuracy drop than RCR for all other reaction types. In the subset in the presence of catalyst, except for the "Reductions and Oxidations ", the accuracies of Parrot and RCR models have the same changes, but the accuracy drop of Parrot in other reaction types are also much less than that of RCR.

**References**

[1] D. Lowe, Chemical reactions from US patents (1976-Sep2016), URL Https://Figshare. Com/Articles/Chemical_ Reactions_from_US_patents_1976-Sep2016_/5104873. (2017). https://doi.org/10.6084/m9.figshare.5104873.v1.

[2] C.W. Coley, W.H. Green, K.F. Jensen, RDChiral: An RDKit Wrapper for Handling Stereochemistry in Retrosynthetic Template Extraction and Application, J Chem Inf Model. 59 (2019) 2529–2537. https://doi.org/10.1021/acs.jcim.9b00286.

[3] G. Landrum, RDKit: Open-source cheminformatics, (n.d.). https://www.rdkit.org (accessed December 30, 2022).

[4] H. Gao, T.J. Struble, C.W. Coley, Y. Wang, W.H. Green, K.F. Jensen, Using Machine Learning to Predict Suitable Conditions for Organic Reactions, ACS Cent Sci. 4 (2018) 1465–1476. https://doi.org/10.1021/acscentsci.8b00357.

[5] M.R. Maser, A.Y. Cui, S. Ryou, T.J. Delano, Y. Yue, S.E. Reisman, Multilabel Classification Models for the Prediction of Cross-Coupling Reaction Conditions, J Chem Inf Model. 61 (2021) 156–166. https://doi.org/10.1021/ACS.JCIM.0C01234/ASSET/IMAGES/LARGE/CI0C01234_0008.JPEG.

[6] P. Schwaller, B. Hoover, J.L. Reymond, H. Strobelt, T. Laino, Extraction of organic chemistry grammar from unsupervised learning of chemical reactions, Sci Adv. 7 (2021). https://doi.org/10.1126/SCIADV.ABE4166/SUPPL_FILE/ABE4166_SM.PDF.

[7] P. Schwaller, D. Probst, A.C. Vaucher, V.H. Nair, D. Kreutter, T. Laino, J.L. Reymond, Mapping the space of chemical reactions using attention-based neural networks, Nature Machine Intelligence 2021 3:2. 3 (2021) 144–152. https://doi.org/10.1038/s42256-020-00284-w.

[8] Https://www.nextmovesoftware.com/pistachio.html, pistachio, (n.d.).

[9] M.D. Driver, C.A. Hunter, Solvent similarity index, Physical Chemistry Chemical Physics. 22 (2020) 11967–11975. https://doi.org/10.1039/D0CP01570A.

[10] B. Zhang, X. Zhang, W. Du, Z. Song, G. Zhang, G. Zhang, Y. Wang, X. Chen, J. Jiang, Y. Luo, Chemistry-informed molecular graph as reaction descriptor for machine-learned retrosynthesis planning, Proc Natl Acad Sci U S A. 119 (2022) e2212711119. https://doi.org/10.1073/PNAS.2212711119/SUPPL_FILE/PNAS.2212711119.SAPP.PDF.

[11] Tanimoto, T. T. An Elementary Mathematical Theory of Classification and Prediction; International Business Machines Corporation, 1958; Google-Books-ID: yp34HAAACAAJ.
